# Flavonoid in All Their Therapeutic Values: An Odyssey into the Phytochemistry and Pharmacology of Naturally Occurring Flavonoid from Genus *Bauhinia*

**DOI:** 10.3390/molecules30163335

**Published:** 2025-08-11

**Authors:** Esther Oluwatosin Shalom, Kolade Olatubosun Faloye, Stephen Adeleke Adesida, Adetola Henry Adewole, Oluwaseun Emmanuel Olatunji, Blessing Ibukun Okunribido, Oluwatosin Funke Olawuni, Esther Aina Olanudun, Seun Bayonle Ogundele, Samson Oluwaseyi Famuyiwa

**Affiliations:** 1 Drug Research and Production Unit, Faculty of Pharmacy, Obafemi Awolowo University, Ile-Ife 220101, Nigeria; 2 Department of Pharmacognosy, Faculty of Pharmacy, Obafemi Awolowo University, Ile-Ife 220101, Nigeria; 3 Department of Industrial Chemistry, Faculty of Sciences, University of Ilesa, Ilesa 5089, Nigeria; 4 School of Biological Sciences, Georgia Institute of Technology, Atlanta, GA 30332, USA; 5 Department of Pharmaceutical Chemistry, Faculty of Pharmacy, Obafemi Awolowo University, Ile-Ife 220101, Nigeria; 6 Department of Chemistry, Faculty of Science, Obafemi Awolowo University, Ile-Ife 220101, Nigeria; 7 Department of Pharmacognosy and Natural Products, College of Pharmacy, Afe Babalola University, Ado-Ekiti 360001, Nigeria

**Keywords:** flavonoid, genus *Bauhinia*, biological activities, classification, structural diversity

## Abstract

The genus *Bauhinia* has over 350 species distributed on different continents of the world. The vast majority of the species in this genus possess interesting biological activities. Also, they are good sources of flavonoids, which are known to elicit excellent pharmacological properties and are well-positioned as potential drug candidates. A literature search was performed with proper consideration of articles published and indexed in PubMed, Scopus, Springer Link, Google Scholar, ScienceDirect, SciFinder, and Medline databases between 1980 and 2023. A total of 164 flavonoids isolated from the genus *Bauhinia* were reviewed, and biological activities including antidiabetic, anti-cancer, antibacterial, cytotoxicity, antidiarrheal, antioxidant, anti-inflammatory, and anti-cataract were all reported. This study gave a comprehensive review of these flavonoids through detailed classification, structural diversity, and pharmacological activities.

## 1. Introduction

Plants comprise 2,500,000 species, while more than 80,000 species have been classified as medicinal, and medicinal chemists have discovered new drug leads from them [1]. The genus *Bauhinia*, classified taxonomically within the Fabaceae family and subfamily Cercidoideae, is an integral component of the huge botanical diversity with an extensive repertoire of approximately 360 species [2,3]. Commonly referred to as the orchid tree or mountain ebony, *Bauhinia* thrives extensively in tropical and subtropical regions, attaining a stature ranging from 6 to 12 m [4]. The species of *Bauhinia* are characterized by flowers with dimensions spanning 7.5 to 12.5 cm, and these blossoms exhibit a spectrum of hues encompassing red, pink, yellow, orange, and purple [3].

*Bauhinia*’s seeds have garnered consumption practices within selected Indian communities owing to their substantial content of proteins, lipids, carbohydrates, and fibers [5]. Notably, the leaves of *Bauhinia* have gained recognition for their efficacy in the management of diabetes mellitus, inflammation, and pain. These activities are attributed to the presence of flavonoids [6,7]. Pharmacologically, *Bauhinia* sp. engenders a diverse array of activities, including anti-inflammatory, antioxidant, cytotoxic, antimycobacterial, antidiabetic, antidiarrheal, antiviral, antiplatelet, anti-cataract, and acetylcholinesterase properties [8,9,10,11,12].

*Bauhinia* species are a rich source of bioactive flavonoids. They are among the most diverse group of phenolic compounds found in nature and have attracted considerable interest due to the beautiful colors they impact on plants [12]. Generally, flavonoid structures are characterized by the presence of two phenyl rings linked together by three carbon atoms, which may be formed into up to six membered rings [13,14]. Within this realm of flavonoids, two predominant forms exist—aglycones and glycosides [3]. The former lacks sugar moieties, while the latter involves flavonoids bound to sugar molecules. Notably, both flavonoid aglycone and glycoside contribute to the overall medicinal values of *Bauhinia* in that, these flavonoids demonstrated antidiabetic, anti-cancer, antioxidant, anti-inflammatory, and anti-cataract activities [14,15,16,17,18,19]. The search for effective natural medications has witnessed tremendous advancements due to their potential to improve the life expectancy of the world population. Natural sugar derivatives have been identified as potential therapeutic agents against diabetes, genetic disorders, cancer, bacterial infection, and viral infection, owing to their ability to inhibit glycosyltransferases (glycosidic bond formation) or glycosidase hydrolysis (breakdown of glycosidic bond) [20,21].

Previous reviews focused on the biological activities of *Bauhinia* species with no emphasis on the classification of the phytochemicals and their pharmacological properties, while others dwelt extensively on the pharmacological efficacy of some *Bauhinia* species [3,4,6,12]. Notably, a review of the chemical constituents from *Bauhinia* species was published about a decade ago [6]. This current endeavor serves as a comprehensive survey of *Bauhinia* sp. flavonoids documented until the year 2023. It also classified them based on their structural features and provided detailed documentation of the observed biological activities within the existing literature.

## 2. Results

Flavonoids make up an extensive group of secondary metabolites having thousands of structures. They are also reported to have a wide range of biological activities. Flavonoids are biosynthesized via the shikimate pathway [16,22]. They are usually composed of a C6-C3-C6 skeleton; two aromatic rings linked by a three-carbon bridge. They are divided into classes based on their degree of unsaturation and substitution patterns [23,24]. This review documented 164 flavonoids belonging to different classes being isolated from the *Bauhinia* genus. About 40% of the compounds occurred as aglycones, while 60% were elucidated as glycosides.

Generally, a total of 76 flavonoid aglycones and 88 flavonoid glycosides comprising various flavonoid subclasses were collated. Among the flavonoid aglycones identified in the *Bauhinia* genus, kaempferol (**1**) and quercetin (**7**) had the highest occurrence of flavonols. Of these flavonols, 6-C-methylquercetin-3,4′-dimethyl ether (**13**) was reported from *B*. *thonningii* for the first time from natural sources. Flavanols like catechin and fisetinidol had the highest occurrence in their sub-class, while naringenin (**32**) and liquiritigenin (**36**), eriodictyol (**39**), and farrerol (**41**) occurred most among flavanones. Out of the 11 flavanones isolated from the genus, 6-butyl-3-hydroxyflavanone was reported as a novel compound from *B. purpurea* [25]. Furthermore, luteolin (**53**), myricetin (**72**), and 5,7,3′,4′,5′-pentamethoxyflavone (**57**) are phytochemicals that occurred mostly among the flavones. Also, bis [3′4′-dihydroxy-6-methoxy-7,8-furano-5′,6′-monomethylallyloxy]-5-C-5-biflavonyl (**76**) was isolated from *B. purpurea*, and these phytochemicals are the two biflavonoid identified in the genus *Bauhinia*.

A total of 88 flavonoid glycosides were purified from the genus *Bauhinia*, out of which rutin (**86**) and quercitrin (**93**) had the highest occurrence. Of the flavonoid glycosides, rhamnetin-3-O-α-L-rhamnopyranosyl-3′-O-(prop-1-enyl) (**137**), a novel propenyl flavonoid glycoside was isolated from Egyptian *B. retusa* [26]. The structural diversity of the phytochemicals and their sources are presented in Table 1, and the structures are illustrated in Table 1 and Figure S1 (see Supplementary Materials).

**Isolation of *Bauhinia* Flavonoids**

Flavonoids from various species of *Bauhinia* are purified using known procedures adopted for other classes of phytochemicals. All the morphological parts are rich in flavonoids. However, the leaves of the *Bauhinia* species have been widely studied compared to other parts. The isolation of flavonoids from the genus involves the use of various chromatographic separation techniques, mainly because these flavonoids exist as aglycones and glycosides. Hence, the isolation of glycosides involves a series of complex approaches due to the sugar attachment.

Generally, the process of isolation and identification of flavonoids from the species involves plant collection and extraction (maceration) processes to obtain flavonoid-rich extract. The extract obtained is fractionated on column chromatography, and the eluates are monitored with thin-layer chromatography using suitable solvent systems. The eluates are further purified to obtain a pure compound that is characterized using nuclear magnetic resonance (NMR) spectroscopy, infrared spectroscopy, and mass spectrometry instruments. Also, secondary metabolite identification methods have been employed to identify flavonoids from the *Bauhinia* genus [67,82].

**Pharmacology**

**Anti-inflammatory activity**

Kaempferol (**1**), ombulin (**4**), kaempferol-3-*O*-β-d-glucopyranoside (**80**), isorhamnetin-3-O-beta-D-glucopyranoside (**79**), and hesperidin (**81**) isolated from the non-woody aerial part of *Bauhinia variegata* were evaluated as inhibitors of some macrophage functions involved in the inflammatory process through lipopolysaccharide (LPS), interferon (IFN)-γ induced nitric oxide (NO), and cytokines [tumor necrosis factor (TNF)-α and interleukin (IL)-12] [16]. These flavonoids inhibited nitrite production induced by LPS/IFN-γ at 5–200 μM significantly in a dose-dependent manner. The addition of isolated phytochemicals to LPS/IFN-γ stimulated peritoneal macrophage cultures effectively inhibited the production of TNF-α and IL-12. Furthermore, the TNF-α and IL-12 production inhibitory assay showed that an inhibition percentage elicited by the compounds ranged from 10 to 30%, indicating that they have good anti-inflammatory activity [16].

In the search for effective anti-inflammatory inhibitors, kaempferol (**1**), ombulin (**4**), and quercetin (**7**) isolated from the methanol extract of *Bauhinia vahlii* were evaluated for in vitro xanthine oxidase inhibitory, cyclooxygenase (COX 1/2), and 5-lipoxygenase inhibitory effects [27]. Quercetin (**7**) exhibited good inhibitory activity against xanthine oxidase (XO) but was not comparable with the reference drug (indomethacin). Also, kaempferol (**1**) and ombuin (**4**) showed significant enzyme COX 1/2 inhibitory effect, while ombuin and quercetin showed the highest 5-LOX inhibitory potential compared to other phytochemicals assayed [34].

Kaempferol (**1**), quercetin (**7**), and kaempferol-3-O-α-L-rhamnoside (**175**) were isolated from *B. retusa* and tested for their efficacy as an anti-inflammatory agent. In the study, NF-kB and iNOS were selected as the inflammatory biomarkers. Kaempferol (**1**) and quercetin (**7**) effectively inhibited iNOS activity with IC_50_ values of 50 and 25 μg/mL, respectively, while no activity was observed against NF-KB. Also, flavonoid glycoside kaempferol-3-*O*-α-L-rhamnoside (**142**) gave the best iNOS inhibitory activity at 19 μg/mL [75]. Similarly, epicatechin gallate isolated from B. hookeri was tested for its efficacy against pro-inflammatory mediators like PGE_2_, TNF-α, IL-1β, and IL-6 in vivo. The results showed that the phytochemical significantly reduced carrageenan-induced paw oedema size by 46, 50, and 58%, respectively. It also elicited reductions in plasma PGE_2_ (27, 38, and 50%), IL-1β (17, 25, and 33%), TNF-α (15, 33, and 41%), and IL-6 (22, 32, and 43%).

**Cytotoxicity activity**

Quercetin (**7**) and isorhamnetin (**73**) isolated from the butanol fraction of *B. foveolata* were evaluated for their cytotoxic activity on human colon cancer cell lines, HT-29 and HCT-15. The results showed that quercetin (**7**) elicited considerably high cytotoxic activity against HT-29 with IC_50_ = 75.33 ± 10.01 µg/mL and HCT-15 with IC_50_ = 105.06 ± 6.52 µg/mL compared to isorhamnetin (**73**), which gave IC_50_ = 115 ± 18.02 µg/mL against HT-29 and IC_50_ = 211 ± 8.02 µg/mL against HCT-15 cell lines. Similarly, Aderogba et al. [79] carried out an antioxidant-guided isolation of flavonoids from *B. tomentosa* and obtained kaempferol-7-*O*-rhamnoside (**162**), kaempferol-3-*O*-glucoside (**163**), quercetin-3-*O*-glucoside (**85**), and quercetin-3-*O*-rutinoside (**150**). The study performed an MTT inhibitory assay on the isolated flavonoid glycosides. Results obtained from the study showed that kaempferol-3-*O*-glucoside (**124**), quercetin-3-*O*-glucoside (**85**), and quercetin-3-*O*-rutinoside (**150**) were non-cytotoxic, while kaempferol-7-*O*-rhamnoside (**162**) were slightly cytotoxic with an IC_50_ value of 116.58 µg/mL [79].

Góis et al. [57] reported that fisetinidol (**24**), (2*R*,3*S*)-2-(3′, 4′-dihydroxyphenyl)-5-methoxychroman-3,7-diol (**21**), and (2*R*,3*S*)-2-(3,4-dihydroxyphenyl)-5-methoxy-6-methylchroman-3,7-diol (**26**), isolated from the roots, and the EtOAc-soluble fraction of the stem wood ethanol extract of *B.ungulata* were evaluated for their pancreas adenocarcinoma (BXPC-3), breast adenocarcinoma (MCF-7), CNS glioblastoma (SF268), lung large cell (NCI-H460), and prostate carcinoma (DU-145) human cancer cell lines inhibitory activities. The results obtained showed that fisetinidol (**24**) was active against all the tested cancer cell lines with IC_50_ < 91.24 µM, (2R,3S)-2-(3′,4′-dihydroxyphenyl)-5-methoxychroman-3,7-diol (**21**) with IC_50_ < 82.24 µM and (2R,3S)-2-(3,4-dihydroxyphenyl)-5-methoxy-6-methylchroman-3,7-diol (**26**) with IC_50_ < 78.62 µM [52].

Tanjung et al. [60] isolated 6C-7O-dimethylaromadendrin (**29**) from the stem bark of *B. semibifida* and assayed the compound for its cytotoxic activity. For the cytotoxic assay, urine leukemia P-388 cells were used. The result obtained from the study showed that 6C-7*O*-dimethylaromadendrin (**29**) gave an impressive cytotoxic activity of 3.98 µg/mL against P-388 cells. In another study conducted on 5,7,4′-Trimethoxy flavanone (**38**) isolated from *B. variegata* against different cancer cell lines of leukemia, non-small cell lung cancer, colon, central nervous system, ovarian, and melanoma using the sulphorhodamine B (SRB) method. The results showed that the flavanone moiety elicited good cytotoxic activity against all the varieties of the tested cell lines [33]. Similarly, a flavonoid glycoside kaempferol-3-*O*-α-L-rhamnoside (**142**) isolated from *B. retusa* was assayed for its cytotoxic activity by evaluating its ability to inhibit LLC-PK1 cells. The phytochemical effectively inhibited LLC-PK1 cell growth with IC_50_ = 19 µg/mL [75].

**Antioxidant activity**

Fisetinidol (**24**) obtained from the ethanol extract of *Bauhinia puchella* was assayed for its antioxidant activity with a focus on DPPH and ABTS assays. The results obtained showed that fisetinidol (**24**) effectively scavenged the radicals with an IC_50_ value of 8.19 mg/mL for DPPH and 7.0237 ± 0.997 mM for the ABTS assay. The IC_50_ value was comparable with alpha-tocopherol used as the reference compound [17].

Epiafzelechin (**23**) and guibourtinidol (**22**) purified from *B. monandra* leaves were evaluated for the radical scavenging potentials using DPPH and FRAPS spectrophotometric methods [58]. The results showed that Epiafzelechin (**23**) gave a good DPPH radical scavenging activity with an IC_50_ value of 269.73 μg/mL, while guibourtinidol (**22**) elicited an IC_50_ value of 1266.89 μg/mL. Kaempferol (**1**) obtained from *B. variegata* stem bark was assayed for its potency as an antioxidant agent using the DPPH and ABTS assays. Kaempferol (1) at a concentration of 20 μg/mL gave 50.51% DPPH and 69.73% ABTS radical scavenging activities [27].

Quercitrin (**93**) and quercetin-3-*O*-β-d-glucopyranosyl-β-d-glucopyranoside (**139**) isolated from *B. retusa* were evaluated for their antioxidant activity by studying their DPPH free radical scavenging property [26]. Quercetirin (**93**) and quercetin-3-*O*-β-d-glucopyranosyl-β-d-glucopyranoside (**139**) gave considerably good radical scavenging activity with IC_50_ values of 13.5 and 14.7 µg/mL. The result obtained for quercitirn (**93**) showed that the flavonoid glycoside elicited better DPPH activity compared to the reference compound (ascorbic acid) that had an IC_50_ value of 13.9 µg/mL.

**Antidiabetic activity**

The alpha glucosidase inhibitory potential of flavonoids isolated from *Bauhinia pottsii* was evaluated in vitro and measured using the colorimetric method. Quercetin (**7**) and 3-O-methyl quercetin (**10**) inhibited the action of the diabetes enzyme with IC_50_ values of 0.486 and 0.292 mM [53]. Epicatechin (**18**), guibourtinidol (**22**), and fisetinidol (**24**) isolated from *B. pulchella* elicited good inhibitory activity against extra-pancreatic diabetes enzymes like alpha glucosidase at 3.62, 0.51, and 0.74 μg/mL in vitro [17].

Praparatana et al. [55] carried out an in vitro antidiabetic study on quercetin (**7**) isolated from the antidiabetic fraction of *B. strychnifolia* extract by considering alpha glucosidase, dipeptidyl peptidase IV, and glucose uptake assays. For alpha glucosidase and dipeptidyl peptidase IV assays, the enzyme and substrates were prepared. Quercetin (**7**) was added, and the absorbance was measured using a microplate reader. The glucose uptake assay was performed by adding quercetin (**7**) to differentiated adipocytes and absorbance measured with a microplate reader. The results indicated that quercetin (**7**) gave good alpha glucosidase and dipeptidyl peptidase inhibitory activities with an IC_50_ value of 6.26 ± 0.36 µM and 8.25 µM. Furthermore, the phytochemical analysis elicited substantial enhancement in glucose uptake focused on 3T3-L1 adipocytes [55]. 

In another antidiabetic study conducted on chemical constituents isolated from *B. pulchella*, the alpha glucosidase inhibitory potential of fisetinidol (**24**) and guibourtinidol (**22**) was evaluated using an in vitro approach. Both fisetinidol (**24**) and guibourtinidol (**22**) gave excellent inhibitory activity with IC_50_ values of 0.51 and 3.67 µg/mL, which are better than acarbose (positive control) that elicited an IC_50_ value of 45.72 µg/mL [17]. Additionally, flavonoid aglycone and flavonoid glycosides were isolated from the fresh leaves of *Bauhinia megalandra* [38], and the compounds were tested for their inhibitory effect against the glucose-6-phosphate (G-6-Pase) enzyme using an in vitro approach. The results showed that quercetin-3-O-alpha-(2″-galloyl)rhamnoside (**158**) and kaempferol-3-*O*-alpha-(2″galloyl)rhamnoside (**157**) had the best inhibitory effect with IC_50_ values of 0.93 ± 0.27 and 1.02 ± 0.22 mM, respectively, while their corresponding aglycones (quercetin (**7**) and kaempferol (**1**)) gave lower inhibitory activity with IC_50_ values of 2.71 ± 0.49 and 2.43 ± 0.05 mM.

The inhibition of glucose intestinal absorption is a unique strategy to manage diabetes in human subjects. Kaempferol-3-*O*-α-rhamnoside (**161**) and quercetin-3-O-α-rhamnoside (**91**) were isolated from *B. megalandra* and evaluated for their ability to inhibit glucose intestinal absorption in vitro at a concentration of 5 mM. The results obtained showed that kaempferol-3-*O*-α-rhamnoside (**161**) inhibited glucose intestinal absorption by 28%, while quercetin-3-*O*-α-rhamnoside (**91**) gave 10% inhibition [30,38].

**Antibacterial activity**

Nouemsi et al. [51] reported 6-*C*-methylquercetin-3,4′-dimethyl ether (**11**) strongly inhibited the growth of *Klebsiella pneumoniae* and *Staphylococcus aureus*. Antibacterial activity of the compound as well as the reference antibiotic (ciprofloxacin) was tested on Gram-negative multidrug-resistant bacteria overexpressing active efflux pumps and methicillin-resistant strains of *Staphylococcus aureus*. Samples were tested alone and in combination with an efflux pump inhibitor (EPI), phenylalanine-arginine-β-naphthylamide (PAßN). Results show that when 6-C-methylquercetin-3,4′-dimethyl ether (**11**) was tested alone, its inhibitory effects were obtained on over two-thirds of the tested bacteria, with the highest MIC value of 128 µg/mL, whilst in the presence of EPI, the growth of all the tested bacteria was inhibited effectively [61]. In another study conducted on narigenin (**31**) and luteolin (**53**) against *B. subtilis*, both flavonoid aglycones gave strong antibacterial activity with more than 50% inhibitory growth of the bacteria [60].

The antibacterial efficacy of flavonoids obtained from *B. thonningii* was evaluated by determining the MIC and MBC through the broth micro-dilution method. Quercetin-3-*O*-L-rhamnopyranoside (**153**) gave good activity of MIC ≤ 32 μg/mL against 50% of the bacteria tested and excellent activity with an MIC value of ≤32 μg/mL against *Pseudomonas aeruginosa* PA124. Furthermore, quercetin-3-*O*-L-rhamnopyranoside (**153**) gave an exponential bacteria growth inhibition as it induced bacteriolysis and completely inhibited the H+-ATPase pumps in *Pseudomonas stuartii* NEA16 [54].

Nguyen et al. [35] investigated the antimyobacterial activities of kaempferol (**1**), ombuin (4), and quercetin (**1**). The results of the antimycobacterial screening revealed that ombulin (**4**) showed better mycobactericidal activity than quercetin (**7**) and kaempferol (**1**) under ex vivo conditions, with minimum inhibitory concentration (MIC) values ranging from 0.05 ± 0.01 to 0.26 ± 0.01 nM and half-maximal inhibitory concentration (IC_50_) values ranging from 2.85 ± 0.14 to 7.21 ± 1.09 nM against dormant and active forms, respectively. Ombulin (**4**) showed higher resistance with MIC values > 100 μg/mL against both gram-positive and gram-negative bacteria and the least cytotoxicity up to 100 μg/mL concentration against the tested series of cancer cell lines [35]. 

Athikomkulchai et al. [61] carried out extensive isolation of flavonoids (naringenin (**32**), luteolin (**53**), and eriodictyol (**39**)) from the stem and root barks of *B. sirindhorniae.* These flavonoids were assayed for their antibacterial activity against *Bacillus subtilis*. Both naringenin (**32**) and luteolin (**53**) gave good antibacterial activity against *Bacillus subtilis* with MIC values of 100 and 200 μg/mL and MBC values > 200 and 200 μg/mL, respectively [61].

**Antiviral activity**

The in vitro antimayaro virus activity of quercetin (**7**) isolated from *B. longifolia* was evaluated by introducing quercetin and ribavirin (standard drug) into 24-well plates. Quercetin (**7**) gave a very potent inhibitory activity of over 90% at 25 μg/mL [46].

**Acetylcholinesterase activity**

Da Silva et al. [18] isolated various sub-classes of flavonoids from the stems of *Bauhinia pentandra* and assayed for their acetylcholinesterase activity using the Ellmann method. The results showed that naringenin (**32**), fisetin (**68**), and 7,4′-dihydroxyflavone (**69**) gave acetycholinesterase inhibitory activity of 0.7, 0.7, 0.9, 0.7, and 0.6 halo diameters (cm), respectively [18].

**Anti-cataract activity**

Bodakhe et al. [10] isolated a flavonol from the stem bark of *B.variegata* and evaluated its anti-cataract activity. The oxidation-antioxidant equilibrium assay was conducted using the ovine and chick embryo lens models. In the study, rhamnocitrin (**9**) was administered at 10, 20, 40, and 80 μg, respectively. The results showed that rhamnocitrin (**9**) gave a dose-dependent activity such that significant lens protection was observed against cloudiness caused by hydrogen peroxide [10].

**Anti-cancer activity**

Zhang et al. [19] isolated dihydrokaempferol (**27**) from *Bauhinia* championii and tested it for its anti-cancer properties with a focus on caspase-9, caspase-3, PARP, and synoviocyte proliferation. Dihydrokaempferol (**27**) exhibited inhibitory effects on the proliferation of synoviocytes. Furthermore, the compound promoted Bax and Bad expression, as well as the cleavage of caspase-9, caspase-3, and PARP. Meanwhile, it inhibited Bcl-2 and Bcl-xL expression. The findings indicated that dihydrokaempferol (**27**) isolated from the ethyl acetate extract of *B. championii* effectively promoted apoptosis, which is an important process through the suppression of apoptotic activity [19].

Yuenyongsawad et al. [42] studied the anti-cancer property of flavonoid glycosides from *B. strychnifolia* by adopting the Sulforhodamine B (SRB) assay for HT-29, HeLa, MCF-7, and KB cell lines. 3,5,7,3′,5′-pentahydroxyflavanonol-3-*O*-α-L-rhamnopyranoside (**88**) and found it to be very active against MCF-7 (IC_50_ = 0.0585 μg/mL), HT-29 (IC_50_ = 0.00217 μg/mL), and HeLa cells (IC_50_ = 0.0692 μg/mL). The phytochemical test showed better KB inhibitory activity against KB (IC_50_ = 0.00054 μg/mL) compared to the anti-cancer drug camptothecin (IC_50_ = 0.0057 μg/mL) [42].

**Analgesic activity**

Hot plate method is among the effective assays carried out when evaluating the analgesic activity of extractives obtained from medicinal plants. Epigallo-catechin-3-*O*-gallate (**94**) isolated from *B. hookeri* was evaluated for its analgesic activity at 100, 200, and 400 mg/kg doses. The results obtained showed that the compound showed good analgesic activity with 32, 52, and 62% inhibition percentages. Also, the activity was dose-dependent [68].

## 3. Materials and Methods

All information on flavonoids from the genus *Bauhinia* was retrieved from PubMed, Scopus, Springer Link, Google Scholar, ScienceDirect, SciFinder, and Medline databases by considering articles published between 1980 and 2023. Search terms like ‘*Bauhinia* flavonoids’, ‘*Bauhinia* flavonoid glycosides’, ‘flavonoids from *Bauhinia*’, ‘chemical constituents from *Bauhinia*’, ‘isolation and characterization of flavonoids from *Bauhinia*’ and therapeutic potential of flavonoids from *Bauhinia*’ were used to select the relevant articles used for the review. The search results obtained were carefully examined for detailed reports of flavonoid aglycones and glycosides with respect to the morphological part(s) used, method of extraction, solvent of extraction as well as their biological activity where applicable.

## 4. Conclusions

This study reviewed the flavonoids previously identified from the genus *Bauhinia* and gave detailed classification as well as their reported pharmacological activities. A total of 164 flavonoids comprising of 76 flavonoid aglycone and 88 flavonoid glycoside moieties were identified from the genus. Flavones dominated the sub-division of flavonoid aglycone isolated from the species, followed by flavonols, while biflavonoids showed the least occurrence. Also, flavonoid glycosides formed the highest number of flavonoids obtained from the genus. The flavonoids aglycones comprised mainly *O*-substitution, C-methylation, and very few prenylated patterns, while the flavonoid glycosides possessed glycosidic linkages and carbohydrate (D-glucose, L-rhamnose, and glucorhamnose) attachments at C-3, C-4′, C-6, C-5, C-7, and C-8 positions. In terms of biological activity, the isolated flavonoids demonstrated promising antidiabetic, antioxidant, anti-inflammatory, anti-cataract, cytotoxic, acetylcholinestrase, antimycobacterial, antibacterial, antidiarrheal, and anti-cancer potentials. Furthermore, the review reported that some of the bioactive flavonoids possess different mechanisms of action against the target diseases, such as antidiabetic, anti-cancer, and antioxidant among others. This review may lead to a robust investigation into the pharmacological potentials and structure-activity relationship study of flavonoid and other chemical constituents obtained from the genus *Bauhinia* to discover potential drug leads in the treatment of communicable and non-communicable diseases.

## Figures and Tables

**Table 1:** Classes, sources, solvent of extraction, and extraction method of flavonoids isolated from Genus *Bauhinia*.

No/Classification	Phytochemical	Source/Solvent of Extraction/Extraction Method	References
1. Flavonol			
	1. Kaempferol	*Bauhinia variegata*/aerial part/methanol/maceration	[16]
		*Bauhinia variegata*/stem bark/80% methanol/maceration	[27]
		*Bauhinia variegata*/stems/methanol/hot extraction	[28]
		*Bauhinia purpurea*/heartwood/methanol/maceration	[29]
		*Bauhinia racemosa*/70% methanol/aerial part/maceration	[30]
		*Bauhinia championii*/rattans	[31]
		*Bauhinia rufescens*/leaves/aqueous acetone/maceration	[32]
		*Bauhinia forficata*/leaves/80% ethanol/maceration	[33]
		*Bauhinia vahlii*/whole plant/90% methanol/maceration	[34]
		*Bauhinia vahlii*/bark/90% methanol/maceration	[35]
		*Bauhinia scandens*/whole plant/ethanol/maceration	[36]
		*Bauhinia glauca*/trunk and branches/70% ethanol/maceration	[37]
		*Bauhinia megalandra*/leaves/methanol/maceration	[38]
	2. 6-methylkaempferol	*Bauhinia glauca*/trunk and branches/70% ethanol/maceration	[37]
	3. 6,8-*C*-dimethyl kaempferol-3-methyl ether	*Bauhinia malabarica*/leaves/methanol/maceration	[39]
	4. Ombuin	*Bauhinia variegata*/aerial part/methanol/maceration	[16]
		*Bauhinia vahlii*/whole plant/90% methanol/maceration	[34]
		*Bauhinia vahlii*/bark/90% methanol/maceration	[35]
	5. 3-hydroxy-6-methoxy-2-phenyl-4H-chromen-4-one	*Bauhinia variegata*/flower/95% ethanol/maceration	[40]
	6. 6-methyl-2-phenyl-4H-chromen-4-one.	*Bauhinia variegata*/flower/95% ethanol/maceration	[40]
	7. Quercetin	*Bauhinia vaeriegata*/aerial part	[41]
		*Bauhinia variegata*/stems/methanol/hot extraction	[28]
		*Bauhinia monandra*/leaves	[32]
		*Bauhinia strychnifolia*/stem/ethanol/maceration	[42]
		*Bauhinia racemosa*/70% methanol/aerial part/maceration	[30]
		*Bauhinia monandra*/leaves	[43]
		*Bauhinia pulla*/leaves/ethanol/maceration	[44]
		*Bauhinia championii*/rattans	[31]
		*Bauhinia galpinii*/leaves/acidified 70% acetone/maceration	[11]
		*Bauhinia ungulata*/leaves/ethanol/maceration	[39]
		*Bauhinia rufescens*/leaves/aqueous acetone/maceration	[45]
		*Bauhinia forficata*/leaves/80% ethanol/maceration	[33]
		*Bauhinia vahlii*/whole plant/90% methanol/maceration	[34]
		*Bauhinia vahlii*/leaves/70% ethanol/maceration	[46]
		*Bauhinia vahlii*/bark/90% ethanol/maceration	[35]
		*Bauhinia longifolia*/leaf/methanol/maceration	[47]
		*Bauhinia scandens*/whole plant/ethanol/maceration	[36]
		*Bauhinia glauca*/aeriel part/85% ethanol/maceration	[48]
		*Bauhinia malabarica*/leaves/methanol/maceration	[39]
		*Bauhinia glauca*/trunk and branches/70% ethanol/maceration	[37]
		*Bauhinia acuminata*/leaves/methanol/maceration	[49]
		*Bauhinia holophylla*/leaves/70% ethanol/maceration	[50]
		*Bauhinia thonningii*/leaves/methanol/maceration	[51]
	8. Quercetin-7-methyl ether	*Bauhinia vaeriegata*/root bark/acetone/maceration	[52]
	9. Rhamnocitrin	*Bauhinia vaeriegata*/stem bark/70% methanol/maceration	[10]
	10. 3-*O*-methylquercetin	*Bauhinia pottsii*/leaves/ethanol/maceration	[53]
	11. 6-*C*-methylquercetin-3, 4′-dimethyl ether	*Bauhinia thonningii*/leaves/methanol/maceration	[51]
	12. 6-*C*-methylquercetin 3,7,3′-trimethyl ether	*Bauhinia thonningii*/leaves/methanol/maceration	[51]
	13. 6-*C*-methylquercetin-3,7-dimethyl ether	*Bauhinia thonningii*/leaves/methanol/maceration	[51]
	14. 6,8-*C*-dimethylquercetin-3-methyl ether	*Bauhinia thonningii*/leaves/methanol/maceration	[51]
	15. 6,8-*C*-dimethylkaempferol-3,7-dimethyl ether	*Bauhinia thonningii*/leaves/methanol/maceration	[51]
	16. 6,8-*C*-dimethylkaempferol-3-methyl ether	*Bauhinia thonningii*/leaves	[54]
	17. 6,8-di-*C*-methylkaempferol 3-methyl ether	*Bauhinia thonningii*/leaves/methanol/maceration	[51]
2. Flavanol			
	18. Epicatechin	*Bauhinia strychnifolia*/stems/95% ethanol/maceration	[55]
		*Bauhinia championii*/	[50]
		*Bauhinia pulchella*/stems/ethanol/maceration	[51]
	19. Catechin	*Bauhinia championii*/	[56]
		*Bauhinia forficata*/leaves/80% ethanol/maceration	[33]
		*Bauhinia scandens*/whole plant/ethanol/maceration	[36]
		*Bauhinia glauca*/trunk and branches/70% ethanol/maceration	[37]
	20. (2*R*,3*S*)-2-(3′,4′-dihydroxyphenyl)-5-methoxy-6-methylchroman-3,7-diol	*Bauhinia acuruana*/roots/ethanol/maceration	[57]
	21. (2*R*,3*S*)-2-(3′,4′-dihydroxyphenyl)-5-methoxychroman-3,7-diol	*Bauhinia acuruana*/roots/ethanol/maceration	[57]
	22. Guibourtinidol	*Bauhinia monandra*/leaves/80% methanol/maceration	[58]
		*Bauhinia ungulata*/roots/ethanol/maceration	[59]
		*Bauhinia pulchella*/stems/ethanol/maceration	[57]
	23. Epiafzelechin	*Bauhinia monandra*/leaves/80% methanol/maceration	[58]
	24. Fisetinidol	*Bauhinia ungulata*/roots/ethanol/maceration	[59]
		*Bauhinia pulchella*/stems/ethanol/maceration	[17]
		*Bauhinia pentandra*/stems	[18]
		Bauhinia acuruana/roots/ethanol/maceration	[57]
	25. -3,4-dihydroxyphenyl-chroman-7-ol	*Bauhinia pulchella*/stems/ethanol/maceration	[17]
	26. (2*R*,3*S*)-2-(3,4-dihydroxyphenyl)-5-methoxy-6-methylchroman-3,7-diol	*Bauhinia acuruana*/roots/ethanol/maceration	[57]
3. Flavononol			
	27. Dihydrokaempferol	*Bauhinia championii*/rattans/95% ethanol/maceration	[19]
	28. Taxifolin	*Bauhinia purpurea*/heartwood/methanol/maceration	[29]
	29. 6*C*-7*O*-dimethylaromadendrin	*Bauhinia semibifida*/stem bark/methanol/maceration	[60]
	30. Garbanzol	*Bauhinia glauca*/aeriel part/85% ethanol/maceration	[48]
4. Flavanone			
	31. 6-butyl-3-hydroxyflavanone	*Bauhinia purpurea*/heartwood/methanol/maceration	[25]
	32. Naringenin	*Bauhinia ungulata*/roots/ethanol/maceration	[59]
		*Bauhinia pentandra*/stems	[18]
		*Bauhinia sirindhorniae*/root and stem/95% ethanol/maceration	[61]
	33. Homoeriodictyol	*Bauhinia glauca*/caulis/95% ethanol/maceration	[62]
	34. 6-methyl-homoeriodictyol	*Bauhinia glauca*/caulis/95% ethanol/maceration	[62]
	35. 3′,4′,7-trihydroxyflavanone	*Bauhinia glauca*/trunk and branches/70% ethanol/maceration	[37]
	36. Liquiritigenin	*Bauhinia championii*/rattans/95% ethanol/maceration	[63]
		*Bauhinia ungulata*/roots/ethanol/maceration	[59]
		*Bauhinia pentandra*/stems	[18]
	37. (2*S*)-5,7-dimethoxy-3′,4′-methylenedioxyflavanone	*Bauhinia vaeriegata*/root bark/acetone/maceration	[64]
	38. 5,7,4′-Trimethoxyflavanone	*Bauhinia vaeriegata*/Stem/90% alcohol/hot extraction	[65]
	39. Eriodictyol	*Bauhinia purpurea*/heartwood/methanol/maceration	[25]
		*Bauhinia ungulata*/roots/ethanol/maceration	[59]
		*Bauhinia sirindhorniae*/root and stem/95% ethanol/maceration	[61]
	40. 5,7,3′,5′-tetrahydroxy-6-methylflavanone	*Bauhinia championii*/rattans/95% ethanol/maceration	[19]
	41. Farrerol	*Bauhinia glauca*/aeriel part/85% ethanol/maceration	[48]
		*Bauhinia glauca*/trunk and branches/70% ethanol/maceration	[37]
5. Flavone			
	42. Chrysin	*Bauhinia purpurea*/bark/methanol/maceration	[66]
	43. 5,7-dihydroxy-3-methoxy-6,8-dimethylflavone	*Bauhinia thonningii*/leaves/methanol/maceration	[51]
	44. 5,7,4′-trihydroxy-3′-methoxyflavone	*Bauhinia glauca*/trunk and branches/70% ethanol/maceration	[37]
	45. 7,3′,4′-trihydroxy-3-methoxyflavone	*Bauhinia glauca*/trunk and branches/70% ethanol/maceration	[37]
	46. 5,7,3′,4′-tetrahydroxy-3-methoxyflavone	*Bauhinia glauca*/trunk and branches/70% ethanol/maceration	[37]
	47. 3,5,7,4′-tetrahydroxy-3′-methoxyflavone	*Bauhinia glauca*/trunk and branches/70% ethanol/maceration	[37]
	48. 3,5,4′-trihydroxy-7-methoxy-6,8-dimethylflavone	*Bauhinia glauca*/trunk and branches/70% ethanol/maceration	[37]
	49. (2*S*)-3′,4′,5,7-tetrahydroxyflavanone	*Bauhinia glauca*/trunk and branches/70% ethanol/maceration	[37]
	50. Strobochrysin	*Bauhinia purpurea*/bark/methanol/maceration	[66]
	51. 5,7-dihydroxy-6,8-dimethylflavone	*Bauhinia purpurea*/bark/methanol/maceration	[66]
	52. 3-methoxy-5,7,3′,4′-tctrahydroxyflavone	*Bauhinia purpurea*/heartwood/methanol/maceration	[25]
	53. Luteolin	*Bauhinia pulla*/leaves/ethanol/maceration	[44]
		*Bauhinia vahlii*/leaves/70% ethanol/maceration	[46]
		*Bauhinia glauca*/aeriel part/85% ethanol/maceration	[48]
		*Bauhinia sirindhorniae*/root and stem/95% ethanol/maceration	[61]
		*Bauhinia holophylla*/leaves/70% ethanol/maceration	[67]
		*Bauhinia hookeri*/leaves/80% ethanol/maceration	[68]
	54. 5-deoxyluteolin	*Bauhinia pulla*/leaves/ethanol/maceration	[44]
	55. 5,6,7,3′,4′,5′-hexanmethoxyflavone	*Bauhinia championii*/rattans/95% ethanol/maceration	[19]
		*Bauhinia championii*/root/ethanol/maceration	[69]
		*Bauhinia championii*	[70]
		*Bauhinia brachycarpa*/leaves/95% ethanol/maceration	[71]
	56. 3′,4′,5,7-tetrahydroxyflavone	*Bauhinia championii*/rattans/95% ethanol/maceration	[19]
	57. 5,7,3′,4′,5′-pentamethoxyflavone	*Bauhinia championii*/rattans/95% ethanol/maceration	[19]
		*Bauhinia championii*/root/ethanol/maceration	[69]
		*Bauhinia brachycarpa*/leaves/95% ethanol/maceration	[71]
	58. 4′-hydroxy-5,7,3′,5′-pentamethoxyflavone	*Bauhinia championii*/rattans/95% ethanol/maceration	[19]
	59. Apigenin	*Bauhinia championii*/rattans/95% ethanol/maceration	[19]
	60. 3′,4′,5,7,8-pentamethoxyflavone	*Bauhinia championii*/rattans/95% ethanol/maceration	[19]
	61. 5,6,7,5′-tetramethoxy-3′,4′-methylenedioxyflavone	*Bauhinia championii*/root/ethanol/maceration	[69]
		*Bauhinia championii*	[70]
		*Bauhinia brachycarpa*/leaves/95% ethanol/maceration	[71]
	62. 5,7,5′-trimethoxy-3′,4′-methylenedioxyflavone	*Bauhinia championii*/root/ethanol/maceration	[69]
	63. 5,6,7,3′,4′-pentamethoxyflavone	*Bauhinia championii*/root/ethanol/maceration	[69]
	64. 5,7,3′,4′-tetramethoxyflavone	*Bauhinia championii*/root/ethanol/maceration	[69]
	65. 5,7,4′,5′ tetrahydroxy-2′-methoxyflavone	*Bauhinia galpinii*/leaves/acidified 70% acetone/maceration	[11]
	66. 3,5,7,2′,4′-pentahydroxyflavone	*Bauhinia tomentosa*/flower bud/chloroform/maceration	[72]
	67. 5-hydroxyflavone	*Bauhinia tomentosa*/flower bud/chloroform/maceration	[72]
	68. Fisetin	*Bauhinia glauca*/aeriel part/85% ethanol/maceration	[48]
		*Bauhinia pentandra*/stems	[18]
	69. 7,4′–dihydroxyflavone	*Bauhinia pentandra*/stems	[18]
	70. 5,6,7,3′,4′,5′-hexamethoxyflavone	*Bauhinia brachycarpa*/leaves/95% ethanol/maceration	[71]
	71. 5,6,7-trimethoxy-3′,4′-methylene-dioxyflavone	*Bauhinia brachycarpa*/leaves/95% ethanol/maceration	[71]
	72. Myricetin	*Bauhinia championii*/rattans	[31]
		*Bauhinia galpinii*/leaves/acidified 70% acetone/maceration	[11]
		*Bauhinia scandens*/whole plant/ethanol/maceration	[36]
	73. Isorhamnetin	*Bauhinia foveolata*/leaves/ethyl acetate and butanol/maceration	[72]
6. Isoflavone			
	74. 7-hydroxy-2-((7-methoxy-2-(4-methoxyphenyl)-4-oxo-4H-chromen-3-yl)methyl)-6-methyl-4H-chromen-4-one	*Bauhinia purpurea*/stem bark/ethanol/hot extraction	[73]
	75. 7-hydroxy-2-[(7-methoxy-4-oxo-4H-chromen-3-yl-2-(4-methoxyphenyl) methyl]-6-methyl-4H-chromen-4-one	*Bauhinia purpurea*/stem bark/ethanol/hot extraction	[73]
7. Biflavonoid			
	76. Bis [3′4′-dihydroxy-6-methoxy-7,8-furano-5′,6′-monomethylallyloxy]-5-C-5-biflavonyl	*Bauhinia purpurea*/70% acetone/maceration	[74]
	77. (4′-hydroxy-7-methyl-3-C-α-Dglucopyranosyl) biflavonoid	*Bauhinia purpurea*/70% acetone/maceration	[74]
8. Flavonoid glycoside			
	78. Kaempferol 7,4′-dimethyl ether 3-*O*-β-d-glucopyranoside	*Bauhinia variegata*/aerial part/methanol/maceration	[16]
		*Bauhinia vaeriegata*/root bark/acetone/maceration	[64]
	79. Isorhamnetin 3-*O*-β-d-glucopyranoside	*Bauhinia variegata*/aerial part/methanol/maceration	[16]
		*Bauhinia vaeriegata*/root bark/acetone/maceration	[64]
	80. Kaempferol 3-*O*-β-d-glucopyranoside	*Bauhinia variegata* aerial part/methanol/maceration	[16]
	81. Hesperidin	*Bauhinia variegata*/aerial part/methanol/maceration	[16]
		*Bauhinia retusa*/flower/ethanol/maceration	[75]
	82. 5-hydroxy7,3′,4′,5′-tetra-methoxyflavone 5-*O*-β-d-xylopyranosyl-(1→2)-α-L-rhamnopyranoside	*Bauhinia variegata*/seeds/95% ethanol/hot extraction	[76]
	83. Naringenin 5,7-dimethyl ether 4′-rhamnoglucoside	*Bauhinia variegata*/stem/ethanol/hot extraction	[77]
	84. Prunin	*Bauhinia variegata*/root/ethanol/hot extraction	[78]
	85. Quercetin 3-*O*-glucoside	*Bauhinia monandra*/leaves	[43]
		*Bauhinia tomentosa*/leaves/50% ethanol/maceration	[79]
	86. Rutin	*Bauhinia monandra*/leaves	[43]
		*Bauhinia monandra*/leaves/methanol/maceration	[80]
		*Bauhinia racemosa*/70% methanol/aerial part/maceration	[30]
		*Bauhinia rufescens*/leaves/aqueous acetone/maceration	[32]
		*Bauhinia rufescens*/leaves/methanol/maceration	[81]
		*Bauhinia forficata*/aerial part/50% methanol/maceration	[82]
		*Bauhinia forficata*/leaves/80% ethanol/maceration	[33]
		*Bauhinia retusa*/flower/ethanol/maceration	[75]
	87. 5,6-dihydroxy-7-methoxyflavone 6-*O*-β-d-xylopyranoside	*Bauhinia purpurea*/stem/ethanol/maceration	[83]
	88. 3,5,7,3′,5′-pentahydroxyflavanonol-3-*O*-α-L-rhamnopyranoside	*Bauhinia strychnifolia*/stem/ethanol/maceration	[42]
	89. Myricetin-3-*O*-β-glucoside	*Bauhinia racemosa*/70% methanol/aerial part/maceration	[30]
	90. kaempferol 3-*O*-β-glucoside	*Bauhinia racemosa*/70% methanol/aerial part/maceration	[30]
	91. quercetin 3-*O*-α-rhamnoside	*Bauhinia racemosa*/70% methanol/aerial part/maceration	[30]
	92. Myricitrin	*Bauhinia championii*	[70]
		*Bauhinia holophylla*/leaves/hydroalcoholic/maceration	[66]
	93. Quercitrin	*Bauhinia pulla*/leaves/ethanol/maceration	[44]
		*Bauhinia championii*	[70]
		*Bauhinia ungulata*/leaves/ethanol/maceration	[45]
		*Bauhinia rufescens*/leaves/aqueous acetone/maceration	[32]
		*Bauhinia vahlii*/leaves/70% ethanol/maceration	[46]
		*Bauhinia retusa*/leaves/ethyl acetate/maceration	[68]
	94. Epigallo-catechin-3-*O*-gallate	*Bauhinia championii*	[56]
		*Bauhinia hookeri*/leaves/80% ethanol/maceration	[68]
	95. Catechin-3-*O*-α-L-rhamnopyranoside	*Bauhinia championii*	[56]
	96. Quercetin-3-*O*-galactopyranoside	*Bauhinia galpinii*/leaves/methanol/maceration	[84]
		*Bauhinia galpinii*/leaves/acidified 70% acetone/maceration	[85]
	97. Myricetin-3-*O*-galactopyranoside	*Bauhinia galpinii*/leaves/methanol/maceration	[84]
		*Bauhinia forficata*/leaves/80% ethanol/maceration	[33]
	98. Myricetin-3-*O*-β-galactopyranoside	*Bauhinia galpinii*/leaves/acidified 70% acetone/maceration	[85]
	99. 2″-*O*-rhamnosylvitexin	*Bauhinia galpinii*/leaves/methanol/maceration	[84]
	100. Hyperoside	*Bauhinia rufescens*/leaves/aqueous acetone/maceration	[32]
		*Bauhinia vahlii*/leaves/70% ethanol/maceration	[46]
	101. Luteolin 4′-*O*-β-d-glucopyranoside	*Bauhinia tarapotensis*/leaves/methanol/maceration	[86]
	102. Kaempferol-3-*O*-rutinoside	*Bauhinia rufescens*/leaves/methanol/maceration	[81]
	103. Kaempferitrin	*Bauhinia forficata*/leaves/marceration	[87]
		*Bauhinia forficata*/leaves/80% ethanol/maceration	[88]
		*Bauhinia forficata*/leaves/methanol	[33]
		*Bauhinia forficata*/leaves/methanol	[89]
	104. Kaempferol-3-*O*-(2-rhamnosyl)glucoside-7-*O*-rhamnoside	*Bauhinia forficata*/aerial part/50% methanol/maceration	[82]
	105. Quercetin arabinofuranoside	*Bauhinia ungulata*/leaves/ethanol/maceration	[45]
	106. Isoquercitrin	*Bauhinia rufescens*/leaves/aqueous acetone/maceration	[32]
		*Bauhinia forficata*/leaves/80% ethanol/maceration	[33]
	107. Kaempferol-3-*O*-(2-rhamnosyl)rutinoside-7-O-rhamnoside	*Bauhinia forficata*/aerial part/50% methanol/maceration	[82]
	108. Myricetin-3-*O*-rhamnoside	*Bauhinia forficata*/leaves/80% ethanol/maceration	[33]
	109. Quercetin-3-*O*-rhamnoside	*Bauhinia forficata*/leaves/80% ethanol/maceration	[33]
	110. Quercetin-3-*O*-galactoside	*Bauhinia forficata*/leaves/80% ethanol/maceration	[33]
	111. 3,7-di-rhamnosyl quercetin	*Bauhinia forficata*/leaves/80% ethanol/maceration	[33]
	112. Quercetin-3-arabinoside	*Bauhinia forficata*/leaves/80% ethanol/maceration	[33]
	113. Kaempferol-3-*O*-glucoside	*Bauhinia forficata*/leaves/80% ethanol/maceration	[33]
		*Bauhinia tomentosa*/leaves/50% ethanol/maceration	[79]
	114. Hyperin	*Bauhinia longifolia*/leaves/methanol/maceration	[47]
		*Bauhinia malabarica*/leaves/methanol/maceration	[39]
		*Bauhinia holophylla*/leaves/hydroalcoholic/maceration	[67]
	115. Guaijaverin	*Bauhinia longifolia*/leaves/methanol/maceration	[47]
	116. Catechin gallate	*Bauhinia longifolia*/leaves/ethanol/maceration	[90]
	117. Kaempferol-deoxyhexose	*Bauhinia longifolia*/leaves/ethanol/maceration	[90]
	118. Juglanin	*Bauhinia longifolia*/leaves/ethanol/maceration	[90]
	119. Avicularin	*Bauhinia longifolia*/leaves/ethanol/maceration	[90]
		*Bauhinia vahlii*/leaves/70% ethanol/maceration	[46]
	120. Quercetin-3-*O*-rutinoside-7-*O*-rhamnoside	*Bauhinia forficata*/aerial part/50% methanol/maceration	[82]
	121. Kaempferol-3-*O*-rutinoside-7-*O*-rhamnoside	*Bauhinia forficata*/aerial part/50% methanol/maceration	[82]
	122. Quercetin-3,7-di-*O*-rhamnoside	*Bauhinia forficata*/aerial part/50% methanol/maceration	[82]
	123. Kaempferol-3-*O*-robinoside	*Bauhinia forficata*/aerial part/50% methanol/maceration	[82]
	124. Isorhamnetin-3-*O*-rutinoside	*Bauhinia forficata*/aerial part/50% methanol/maceration	[82]
	125. Myricetin-3-*O*-hexoside	*Bauhinia forficata*/aerial part/50% methanol/maceration	[82]
	126. Myricetin-3-*O*-xyloside	*Bauhinia forficata*/aerial part/50% methanol/maceration	[82]
	127. Myricetin-3-*O*-arabinofuranoside	*Bauhinia forficata*/aerial part/50% methanol/maceration	[82]
	128. Quercetin-3-*O*-xyloside	*Bauhinia forficata*/aerial part/50% methanol/maceration	[82]
	129. Kaempferol-3,7-di-*O*-α-L-rhamnoside	*Bauhinia retusa*/leaves/50% ethanol/maceration	[91]
	130. Quercetin-3-7-di-*O*-α-L-rhamnoside	*Bauhinia retusa*/leaves/50% ethanol/maceration	[91]
	131. Kaempferol-3-*O*-α-L-rhamnosyl (1→2)-β-d-glycoside	*Bauhinia retusa*/leaves/50% ethanol/maceration	[91]
	132. Quercetin-3-*O*-β-d-glucosyl (1→2)-β-d-galactoside-7-*O*-β-d-glucoside	*Bauhinia retusa*/leaves/50% ethanol/maceration	[91]
	133. Kaempferol-3-*O*-α-L-rhamnoside (1→6)-β-glucopyranoside-7-*O*-β-d-glucoside	*Bauhinia retusa*/leaves/50% ethanol/maceration	[91]
	134. Quercetin-3-*O*-α-L-rhamnosyl (1→6)-β-galucopyranoside-7-*O*-β-d-glucoside	*Bauhinia retusa*/leaves/50% ethanol/maceration	[91]
	135. Isorhamnetin-3-*O*-α-L-rhamnopyranosyl (1→2)-β-glactopyranoside-7-*O*-β-d-glucoside	*Bauhinia retusa*/leaves/50% ethanol/maceration	[91]
	136. Kaempferol-3-*O*-β-glucosyl (1→2)-α-rhamnosyl (1→6)-β-d-glucopyranoside-7-*O*-α-L-rhamnoside	*Bauhinia retusa*/leaves/50% ethanol/maceration	[91]
	137. Rhamnetin-3-*O*-α-rhamnopyranosyl-3′-*O*-(prop-1-enyl)	*Bauhinia retusa*/leaves/90% ethanol/maceration	[26]
	138. Quercitin-3′-methoxy-3-*O*-rhamnoside	*Bauhinia retusa*/leaves/90% ethanol/maceration	[26]
	139. Quercetin-3-*O*-β-d-glucopyranosyl-β-d-glucopyranoside	*Bauhinia retusa*/leaves/90% ethanol/maceration	[26]
	140. Quercetin-3,7-di-*O*-β-d-glucoside	*Bauhinia retusa*/leaves/90% ethanol/maceration	[26]
	141. Quercetin-3-*O*-α-L-rhamnoside	*Bauhinia retusa*/leaves/90% ethanol/maceration	[75]
	142. Kaempferol-3-*O*-α-L-rhamnoside	*Bauhinia retusa*/leaves/90% ethanol/maceration	[75]
	143. Myricetin-3-*O*-α-L-rhamnoside	*Bauhinia retusa*/leaves/90% ethanol/maceration	[75]
	144. Quercetin-3-*O*-α-L-rhamnoside	*Bauhinia retusa*/leaves/90% ethanol/maceration	[75]
	145. Quercetin-3-*O*-β-L-galactoside	*Bauhinia retusa*/leaves/90% ethanol/maceration	[75]
	146. Kaempferol-3-*O*-(6″-*O*-galloyl) β-d-glucoside	*Bauhinia retusa*/leaves/90% ethanol/maceration	[75]
	147. Odoratin-7-glucoside	*Bauhinia foveolata*/leaves/methanol/maceration	[72]
	148. Astragalin-2″,6″-*O*-digallate	*Bauhinia microstachya*/leaves/ethanol/maceration	[92]
	149. Kaempferol-3-*O*-rhamnoside	*Bauhinia microstachya*/leaves/ethanol/maceration	[92]
		*Bauhinia tomentosa*/leaves/50% ethanol/maceration	[79]
	150. Quercetin-3-*O*-rutinoside	*Bauhinia tomentosa*/leaves/50% ethanol/maceration	[79]
	151. 5,7,3′,4′-tetrahydroxyflavone-3-*O*-rhamnoside	*Bauhinia tomentosa*/flowering bud/chloroform/maceration	[93]
	152. 6,8-*C*-dimethyl kaempferol-3-*O*-rhamnopyranoside	*Bauhinia malabarica*/leaves/methanol/maceration	[39]
	153. Quercetin-3-*O*-L-rhamnopyranoside	*Bauhinia thonningii*/leaves/methanol/maceration	[51]
	154. Quercetin-3-*O*-β-glucopyranoside	*Bauhinia thonningii*/leaves/methanol/maceration	[51]
	155. Kaempferol-7,4′-dimethyl ether 3-*O*-β-d-glucopyranoside	*Bauhinia variegata*/aerial part/methanol/maceration	[16]
	156. Quercetin-3-*O*-β-sophoroside	*Bauhinia vahlii*/leaves/70% ethanol/maceration	[46]
	157. kaempferol 3-*O*-alpha-(2″-galloyl)rhamnoside	*Bauhinia megalandra*/leaves/methanol/maceration	[38]
	158. Quercetin 3-*O*-alpha-(2″-galloyl)rhamnoside	*Bauhinia megalandra*/leaves/methanol/maceration	[38]
	159. Quercetin 3-*O*-α-rhamnoside	*Bauhinia megalandra*/leaves/methanol/maceration	[94]
	160. Quercetin-3-*O*-glucoside	*Bauhinia tomentosa*/leaves/50% ethanol/maceration	[79]
	161. Kaempferol-3-*O*-α-rhamnoside	*Bauhinia megalandra*/leaves/methanol/maceration	[94]
	162. Kaempferol-7-*O*-rhamnoside	*Bauhinia tomentosa*/leaves/50% ethanol/maceration	[79]
	163. Kaempferol-3-*O*-glucoside	*Bauhinia tomentosa*/leaves/50% ethanol/maceration	[79]
	164. 6,6-bisastilbin	*Bauhinia aurea*/stems/90% aqueous ethanol/maceration	[95]

## Supplementary Materials

The following supporting information can be downloaded at: https://www.mdpi.com/article/10.3390/molecules30163335/s1, Figure S1: Chemical structures of flavonoids from *Bauhinia* genus.
