# Supplementary material for: Flavonoid in All Their Therapeutic Values: An Odyssey into the Phytochemistry and Pharmacology of Naturally Occurring Flavonoid from Genus Bauhinia
Source: Molecules. 2025 Aug 11;30(16):3335. doi: 10.3390/molecules30163335 (PMC12388531; doi:10.3390/molecules30163335)
Supplement: Supplementary file 1 [file molecules-30-03335-s001.zip › molecules-3035219-supplementary.pdf]

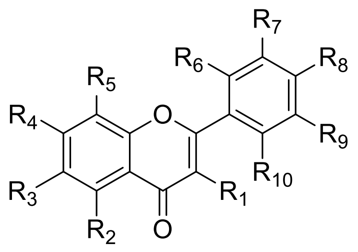

1: R_1=R_2=R_4=R_8=**OH**, R_3=R_5=R_6=R_7=R_9=R_{10}=**H**

2: R_1=R_2=R_8=**OH**, R_4=**CH_3,** R_3=R_5=R_6=R_7=R_9=R_{10}=**H**

3: R_1=**OCH_3,** R_2=R_4=R_8=**OH**, R_3=R_5=**CH_3,** R_6=R_7=R_9=R_{10}=**H**

4: R_1=R_2=R_4=R_7=**OH**, R_8=**OCH_3**, R_3=R_5=R_6=R_7=R_9=R_{10}=**H**

5: R_1= **OH**, R_3=**OCH_3,** R_2=R_4=R_5=R_6=R_7=R_8=R_9=R_{10}=**H**

6: R_1=R_8**=OH**, R_3=**CH_3,** R_2=R_4=R_5=R_6=R_7=R_9=R_{10}=**H**

7: R_1=R_7=R_8**=OH**, R_3=**CH_3,** R_2=R_4=R_5=R_6=R_9=R_{10}=**H**

8: R_1=R_2=R_4=R_7=R_8**=OH**, R_3=**OCH_3,** R_5=R_6=R_9=R_{10}=**H**

9: R_1=R_2=R_8**=OH**, R_4=**OCH_3,** R_3**=**R_5=R_6=R_7=R_9=R_{10}=**H**

10: R_1=**OCH_3,** R_2=R_4=R_7=R_8**=OH**, R_3**=**R_5=R_6=R_9=R_{10}=**H**

11: R_1=R_7=R_8**=OCH_3**, R_1=R_4**=OH**, R_3=**OCH_3,** R_5=R_6=R_9=R_{10}=**H**

12: R_1=R_4=R_9**=OCH_3**, R_2=R_8**=OH**, R_3=**CH_3,** R_5=R_6=R_7=R_{10}=**H**

13: R_1=R_4=**OCH_3,** R_2=R_8=R_9**=OH**, R_3=**CH_3,** R_5=R_6=R_7=R_{10}=**H**

14: R_1=**OCH_3**, R_2=R_4=R_8=R_9**=OH**, R_3=R_5=**CH_3,** R_6=R_7=R_{10}=**H**

15: R_1=R_3=**CH_3,** R_2=R_4=R_8**=OH**, R_5=**OCH_3,** R_6=R_7=R_9=R_{10}=**H**

16: R_1=**OCH_3,** R_2=R_4=R_8**=OH**, R_3**=**R_5=**CH_3,** R_6=R_7=R_9=R_{10}=**H**

17: R_1=R_2=R_8**=OH**, R_3=R_5**=CH_3,** R_4=**OCH_3,** R_6=R_7=R_9=R_{10}=**H**

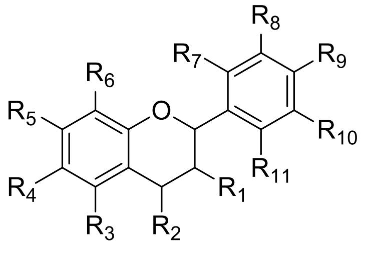

18=19: $R_1=R_3=R_5=R_8=R_9=$**OH**, $R_2=R_4=R_6=R_7=R_{10}=R_{11}=$**H**

20: $R_1=R_5=R_8=R_9=$**OH**, $R_2=R_6=R_7=R_{10}=R_{11}=$**H**, $R_3=$**OCH_3**, $R_4=$**CH_3**

21: $R_1=R_5=R_8=R_9=$**OH**, $R_2=R_4=R_6=R_7=R_{10}=R_{11}=$**H**, $R_3=$**OCH_3**

22: $R_1=R_5=R_8=R_9=$**OH**, $R_2=R_6=R_7=R_{10}=R_{11}=$**H**, $R_3=$**OCH_3**, $R_4=$**CH_3**

23: $R_1=R_3=R_5=R_9=$**OH**, $R_2=R_4=R_6=R_7=R_8=R_{10}=R_{11}=$**H**

24: $R_1=R_5=R_8=R_9=$**OH**, $R_2=R_3=R_4=R_6=R_7=R_{10}=R_{11}=$**H**

25: $R_1=R_2=R_3=R_4=R_6=R_7=R_8=R_{10}=R_{11}=$**H**, $R_5=R_9=$**OH**

26: $R_1=R_5=R_8=R_9=$**OH**, $R_2=R_6=R_7=R_{10}=R_{11}=$**OH**, $R_3=$**OCH_3**, $R_4=$**CH_3**

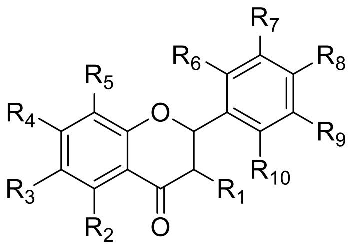

27: $R_1=R_2=R_4=R_8=$**OH**, $R_3=R_5=R_6=R_7=R_9=R_{10}=$**H**

28: $R_1=R_2=R_4=R_7=R_8=$**OH**, $R_3=R_5=R_6=R_7=R_9=R_{10}=$**H**

29: $R_1=R_2=R_8=$**OH**, $R_3=$**CH_3**, $R_3=$**OCH_3**, $R_5=R_6=R_7=R_9=R_{10}=$**H**

30: $R_1=R_4=R_8=$**OH**, $R_2=R_3=R_5=R_6=R_7=R_9=R_{10}=$**H**

31: $R_1=R_2=R_4=R_7=R_8=$**OH**, $R_3=$**$CH_2COCH_2CH_3$**, $R_5=R_6=R_9=R_{10}=$**H**

32: $R_1=R_3=R_5=R_6=R_7=R_9=R_{10}=$**H**, $R_2=R_4=R_8=$**OH**

33: $R_1=R_3=R_5=R_6=R_9=R_{10}=$**H**, $R_2=R_4=R_8=$**OH**, $R_7=$**OCH_3**

34: $R_1=R_5=R_6=R_9=R_{10}=$**H**, $R_2=R_4=R_8=$**OH**, $R_3=$**CH_3**, $R_7=$**OCH_3**

35: $R_1=R_2=R_3=R_5=R_6=R_9=R_{10}=$**H**, $R_4=R_7=R_8=$**OH**

36: $R_1=R_3=R_5=R_6=R_7=R_9=R_{10}=$**H**, $R_4=R_8=$**OH**

38: $R_1=R_3=R_5=R_6=R_7=R_9=R_{10}=$**H**, $R_2=R_4=R_8=$**OCH_3**

39: $R_1=R_3=R_5=R_6=R_9=R_{10}=$**H**, $R_2=R_4=R_7=R_8=$**OH**

40: $R_1=R_5=R_6=R_8=R_{10}=$**H**, $R_2=R_4=R_7=R_9=$**OH**, $R_3=$**CH_3**

41: $R_1=R_6=R_7=R_9=R_{10}=$**H**, $R_2=R_4=R_8=$**OH**, $R_3=R_5=$**CH_3**

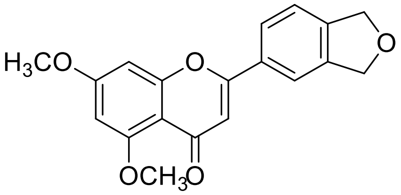

37

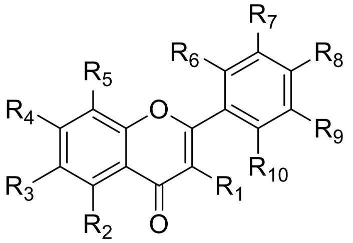

42: $R_1=R_3=R_5=R_6=R_7=R_8=R_9=R_{10}=$**H**, $R_2=R_4=$**OH**

43: $R_1=$**OCH_3**, $R_2=R_4=$**OH**, $R_3=R_5=$**CH_3**, $R_6=R_7=R_8=R_9=R_{10}=$**H**

44: $R_1=R_3=R_5=R_6=R_7=R_{10}=$**H**, $R_2=R_4=R_9=$**OH**, $R_8=$**OCH_3**

45: $R_1=$**OCH_3**, $R_2=R_3=R_5=R_6=R_9=R_{10}=$**H**, $R_4=R_7=R_8=$**OH**

46: $R_1=$**OCH_3**, $R_2=R_4=R_7=R_8=$**OH**, $R_3=R_5=R_6=R_9=R_{10}=$**H**

47: $R_1=R_2=R_4=R_8=$**OH**, $R_3=R_5=R_6=R_9=R_{10}=$**H**, $R_7=$**OCH_3**

48: $R_1=R_2=R_8=$**OH**, $R_3=R_5=$**CH_3**, $R_4=$**OCH_3**, $R_6=R_7=R_9=R_{10}=$**H**

49: $R_1=R_2=R_4=R_7=R_8=$**OH**, $R_3=R_5=R_6=R_9=R_{10}=$**H**

50: $R_1=R_5=R_6=R_7=R_8=R_9=R_{10}=$**H**, $R_2=R_4=$**OH**, $R_3=$**CH_3**

51: $R_1=$**OCH_3**, $R_2=R_4=$**OH**, $R_3=R_5=CH_3$, $R_6=R_7=R_8=R_9=R_{10}=$**H**

52: $R_1=$**OCH_3**, $R_2=R_4=R_8=R_9=$**OH**, $R_3=R_5=R_6=R_7=R_{10}=$**H**

53: $R_1=R_3=R_5=R_6=R_9=R_{10}=$**H**, $R_2=R_4=R_7=R_8=$**OH**

54: $R_1=R_2=R_3=R_5=R_6=R_9=R_{10}=$**H**, $R_4=R_7=R_8=$**OH**

55: $R_1=R_5=R_6=R_{10}=$**H**, $R_2=R_3=R_4=R_7=R_8=R_9=$**OCH_3**

56: $R_1=R_2=R_4=R_8=$**H**, $R_3=R_5=R_6=R_7=R_9=R_{10}=$**H**

57: $R_1=R_3=R_5=R_6=R_{10}=$**H**, $R_2=R_4=R_7=R_8=R_9=$**OCH_3**

58: $R_1=R_3=R_5=R_6=R_{10}=\mathbf{H}$, $R_2=R_4=R_7=R_9=\mathbf{OCH_3}$**,** $R_8=\mathbf{OH}$

59: $R_1=R_3=R_5=R_6=R_7=R_9=R_{10}=\mathbf{H}$, $R_2=R_4=R_8=\mathbf{OH}$

60: $R_1=R_3=R_6=R_7=R_{10}=\mathbf{H}$, $R_2=R_4=R_5=R_8=R_9=\mathbf{OCH_3}$

63: $R_1=R_5=R_6=R_7=R_{10}=\mathbf{H}$, $R_2=R_3=R_4=R_8=R_9=\mathbf{OCH_3}$

64: $R_1=R_3=R_5=R_6=R_7=R_{10}=\mathbf{H}$, $R_1=R_2=R_4=R_8=R_9=\mathbf{OCH_3}$

65: $R_1=R_3=R_5=R_6=R_9=\mathbf{H}$, $R_2=R_4=R_7=R_8=\mathbf{OH}$, $R_{10}=\mathbf{OCH_3}$

66: $R_1=R_2=R_4=R_8=R_{10}=\mathbf{OH}$, $R_3=R_5=R_6=R_7=R_9=\mathbf{H}$

67: $R_1=R_3=R_4=R_5=R_6=R_7=R_8=R_9=R_{10}=\mathbf{H}$, $R_2=\mathbf{OH}$

68 $R_1=R_4=R_7=R_8=\mathbf{OH}$, $R_2=R_3=R_5=R_6=R_9=R_{10}=\mathbf{H}$

69: $R_1=R_2=R_3=R_5=R_6=R_7=R_9=R_{10}=\mathbf{H}$, $R_4=R_8=\mathbf{OH}$

70=71: $R_1=R_5=R_6=R_{10}=\mathbf{H}$, $R_2=R_3=R_4=R_7=R_8=R_9=\mathbf{OCH_3}$

72: $R_1=R_2=R_4=R_7=R_8=R_9=\mathbf{OH}$, $R_3=R_5=R_6=R_{10}=\mathbf{H}$

73: $R_1=R_2=R_4=R_8=\mathbf{OH}$, $R_3=R_5=R_6=R_9=R_{10}=\mathbf{H}$, $R_7=\mathbf{OCH_3}$

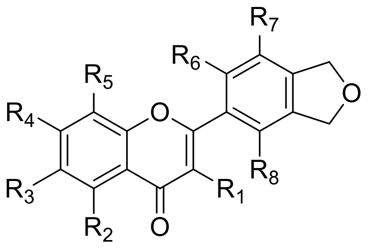

61: $R_1=R_5=R_6=R_8=\mathbf{H}$, $R_1=R_2=R_3=R_4=R_7=\mathbf{OCH_3}$

62: $R_1=R_3=R_5=R_6=R_8=\mathbf{H}$, $R_2=R_4=R_7=\mathbf{OCH_3}$

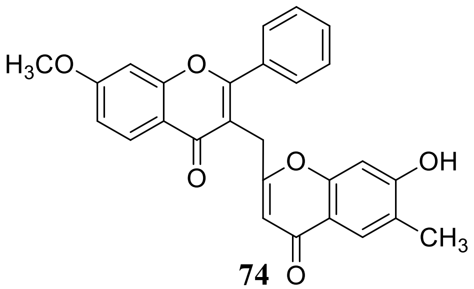

74

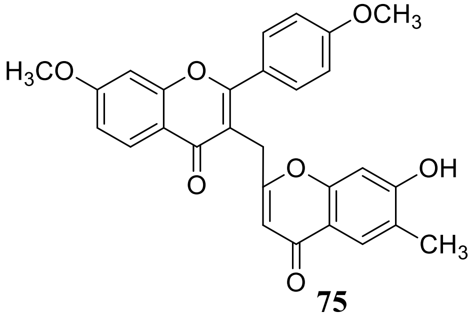

75

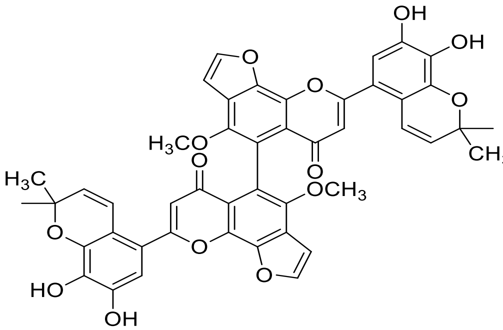

76

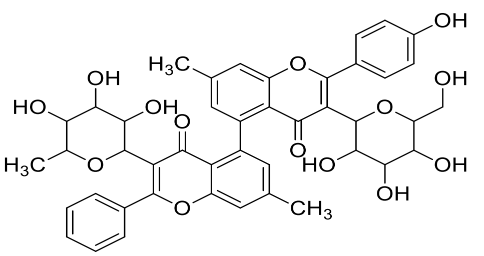

77

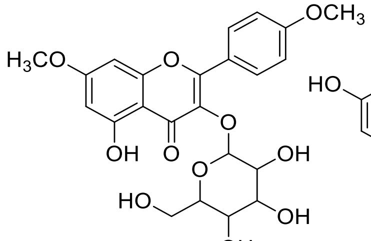

78

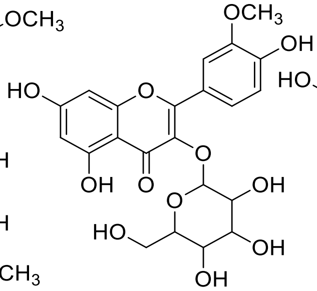

79

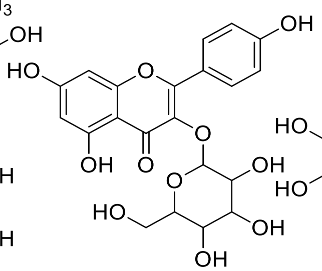

80

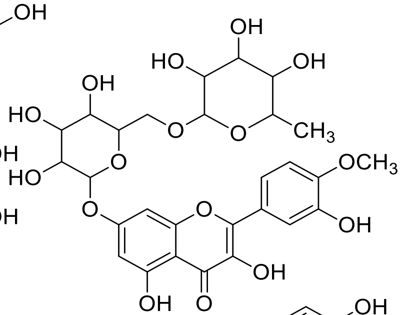

81

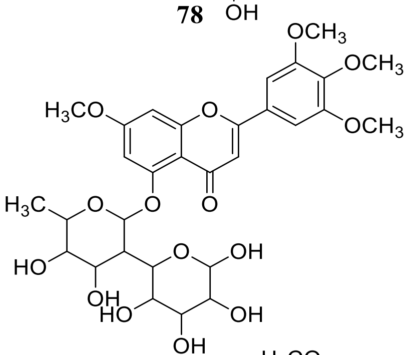

82

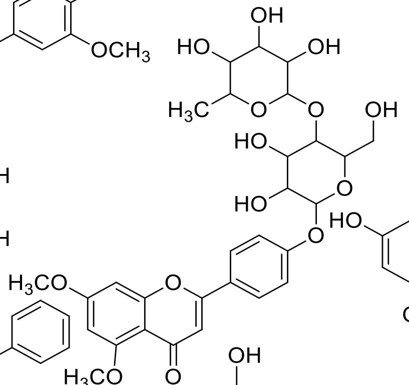

83

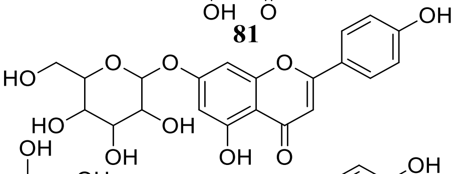

84

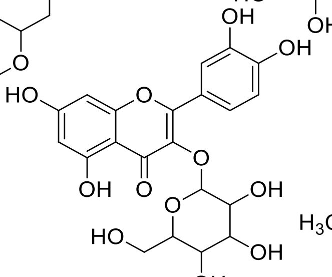

85

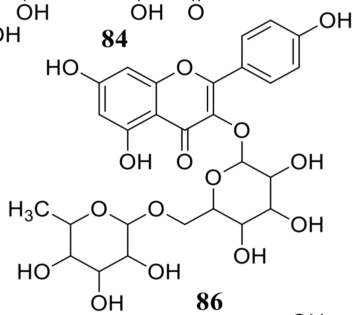

86

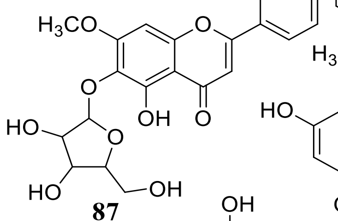

87

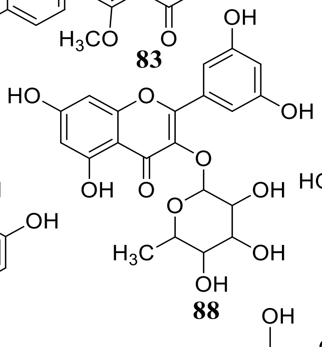

88

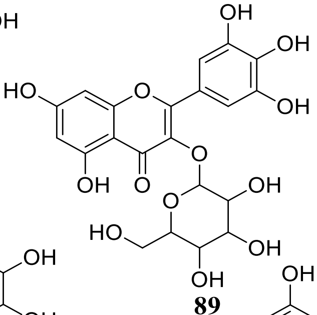

89

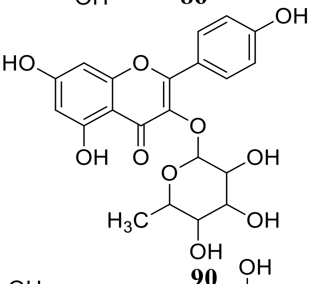

90

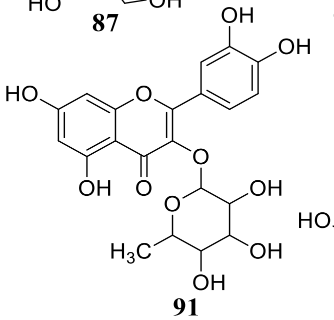

91

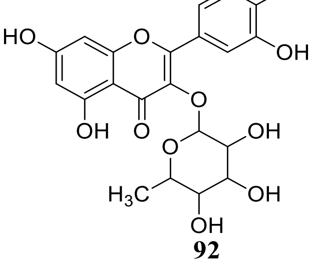

92

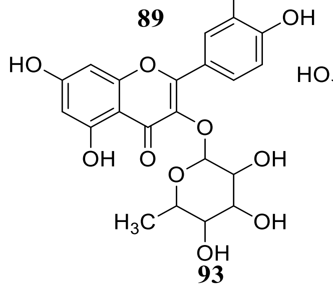

93

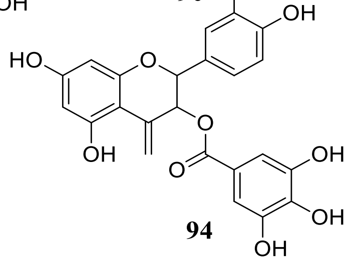

94

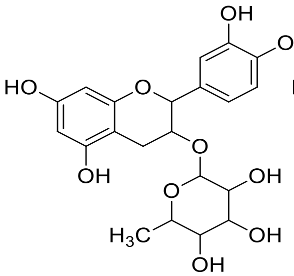

95

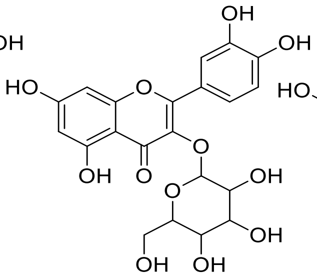

96

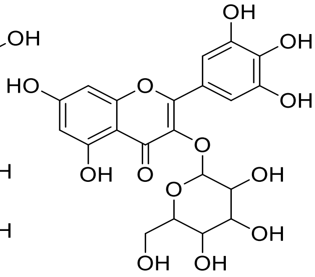

97

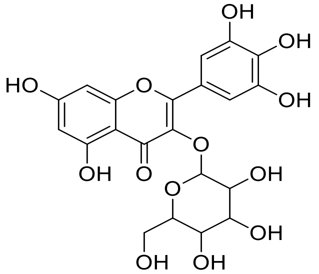

98

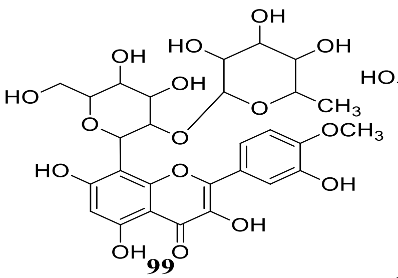

99

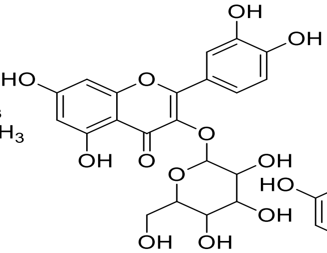

100

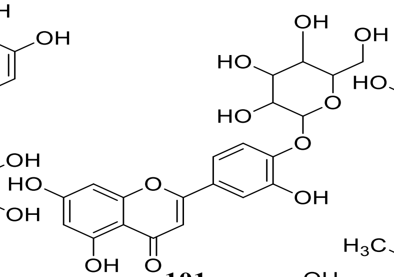

101

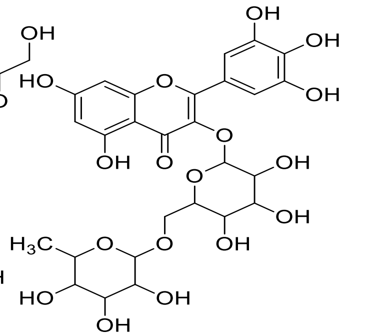

102

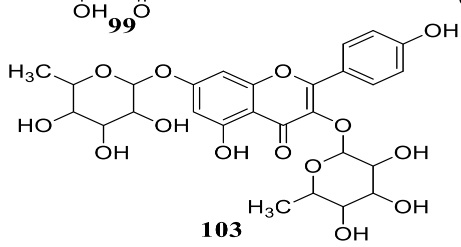

103

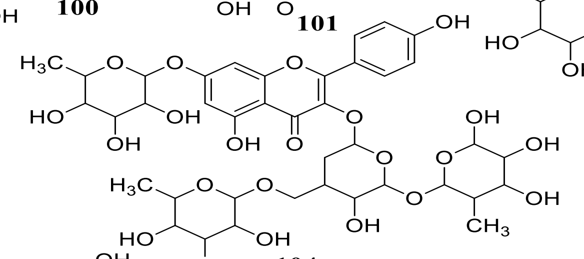

104

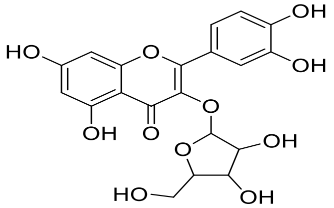

105

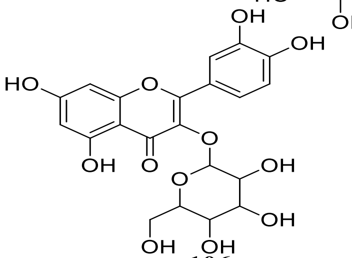

106

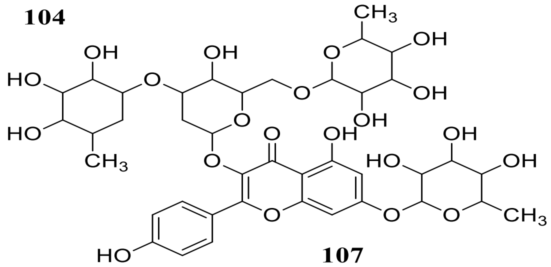

107

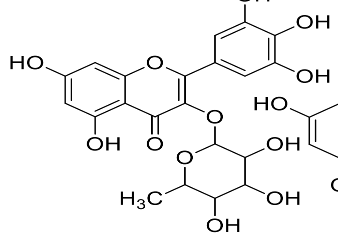

108

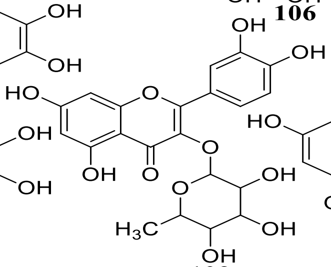

109

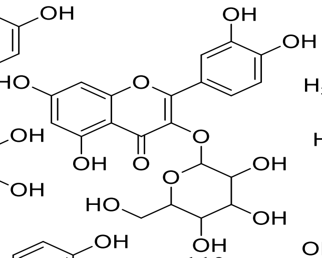

110

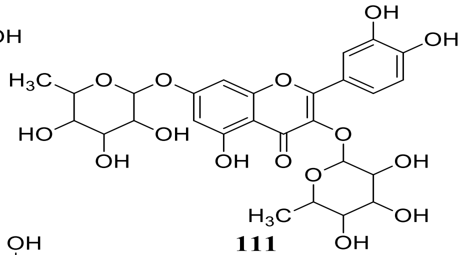

111

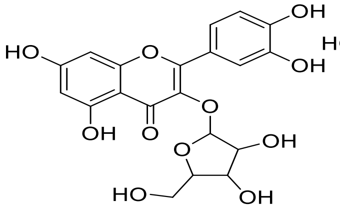

112

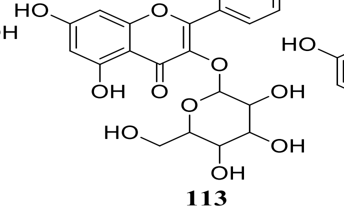

113

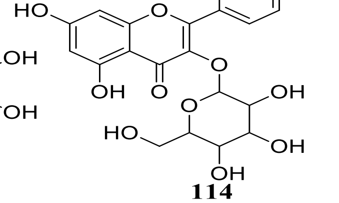

114

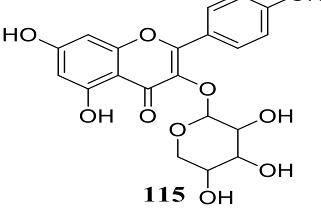

115

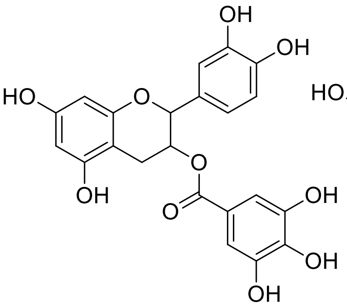
116

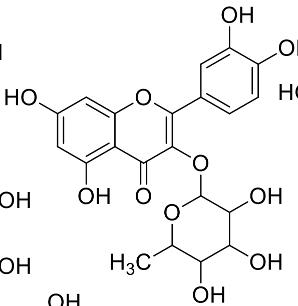
117

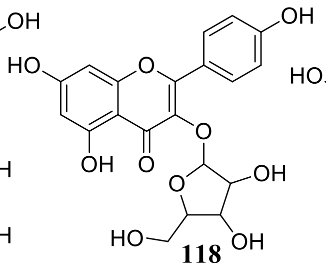
118

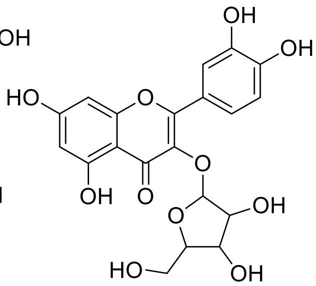
119

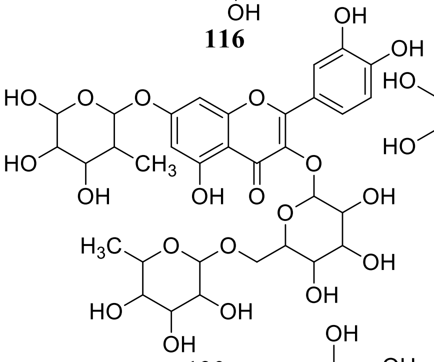
120

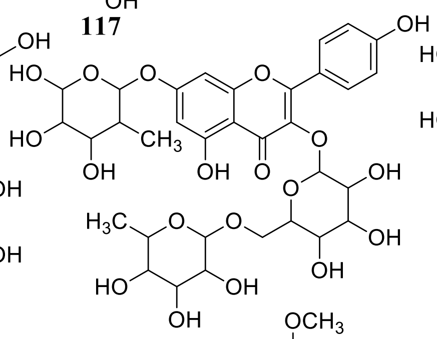
121

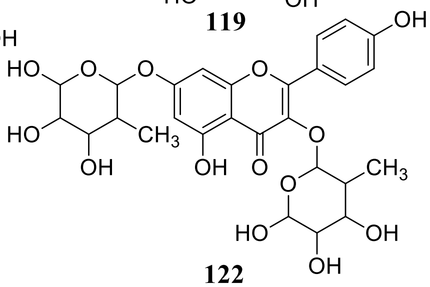
122

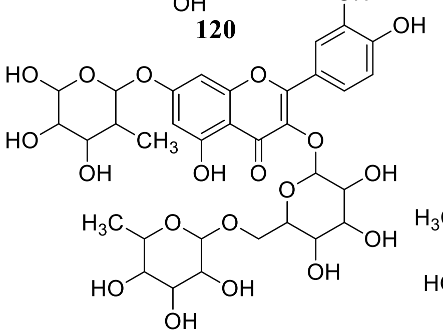
123

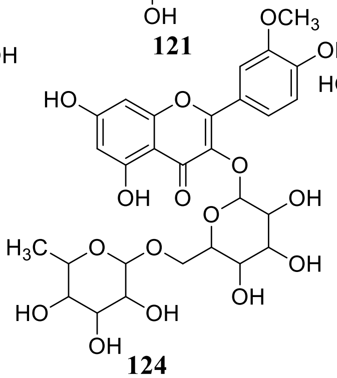
124

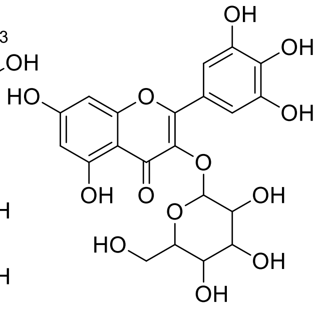
125

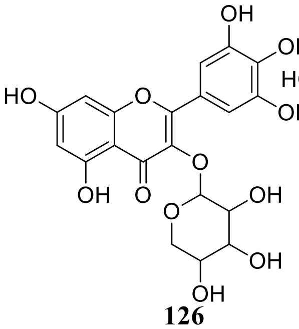
126

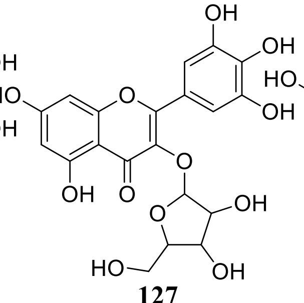
127

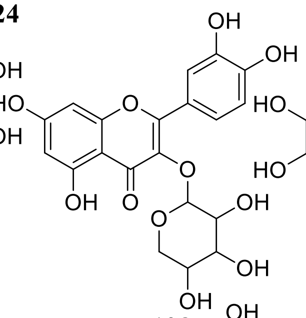
128

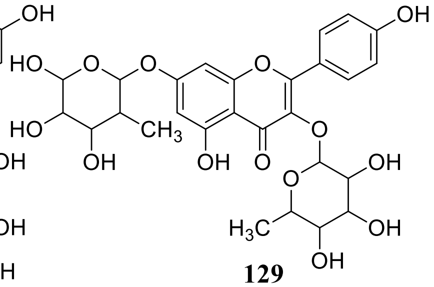
129

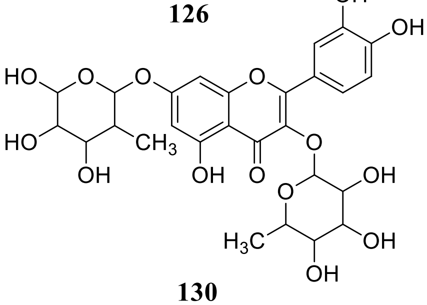
130

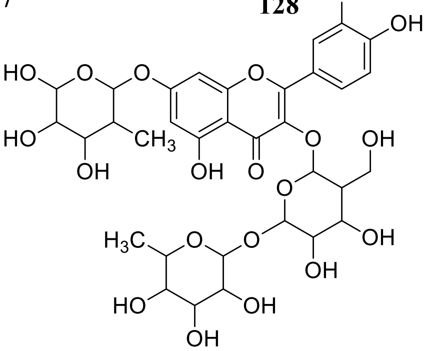
131

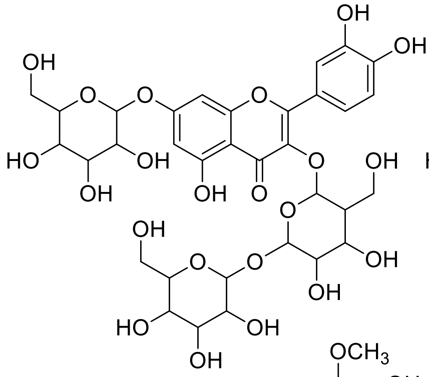

132

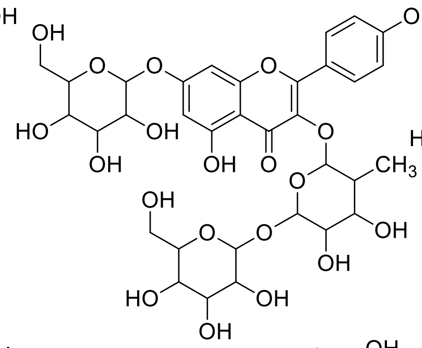

133

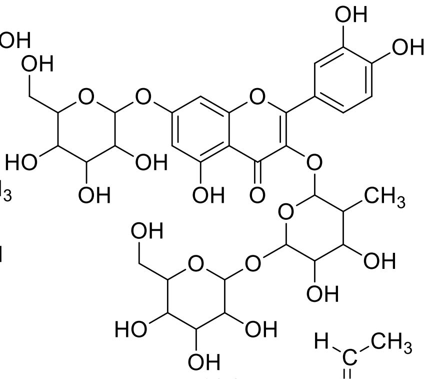

134

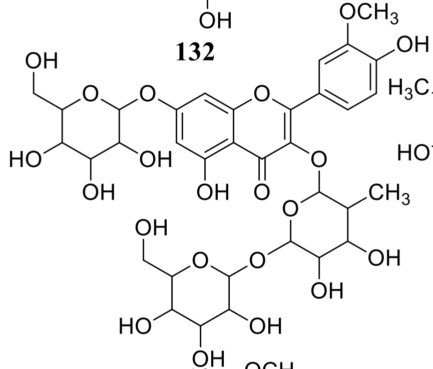

135

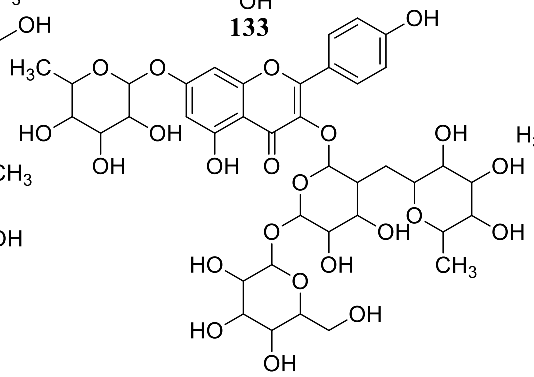

136

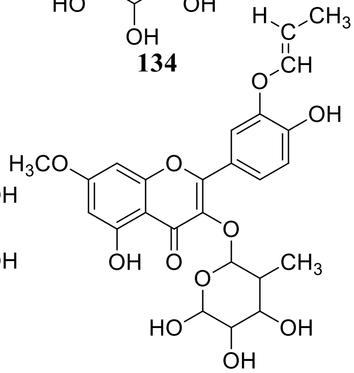

137

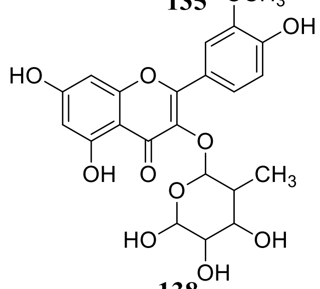

138

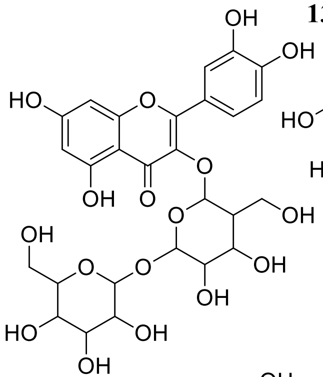

139

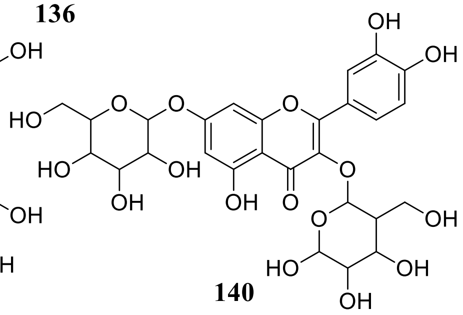

140

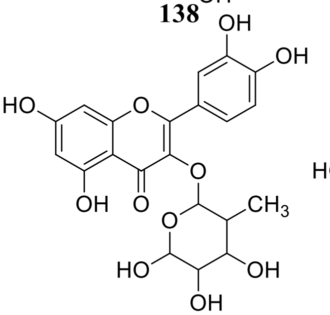

141

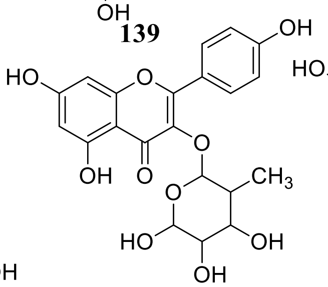

142

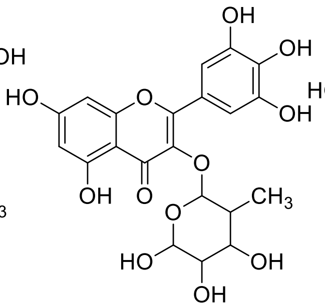

143

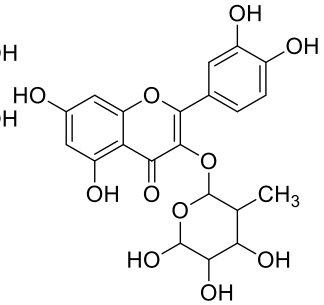

144

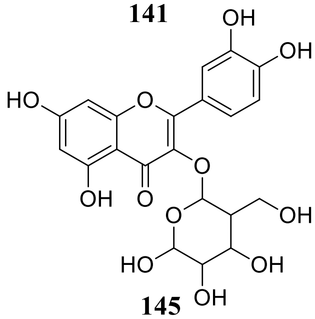

145

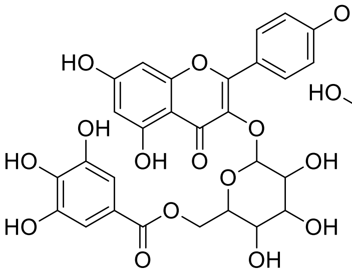
146

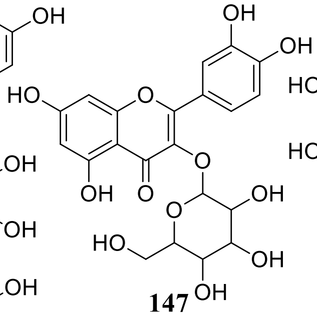
147

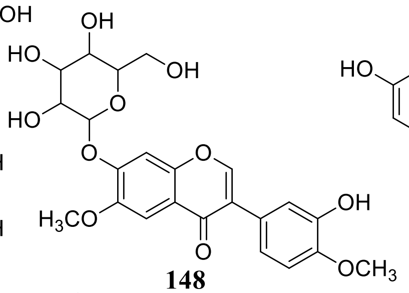
148

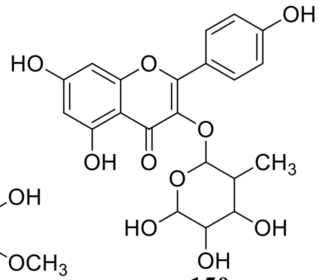
150

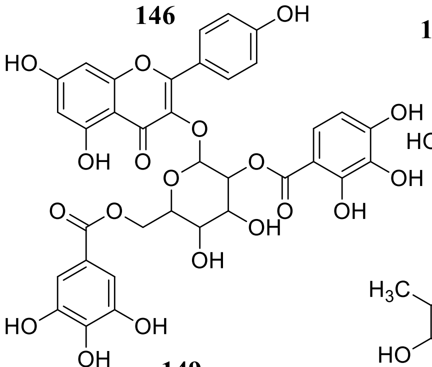
149

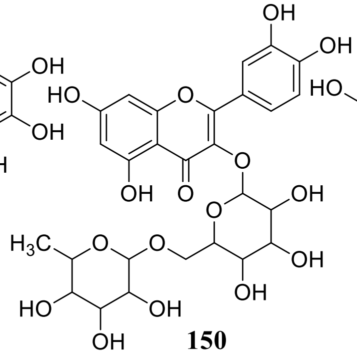
150

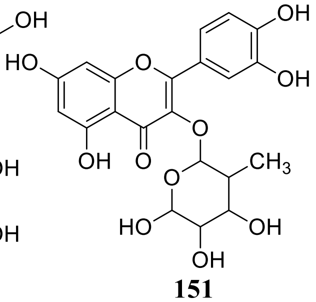
151

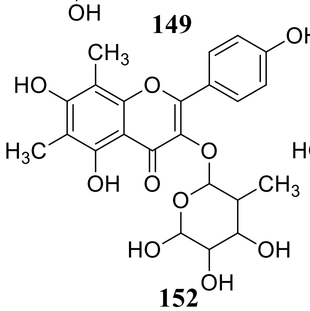
152

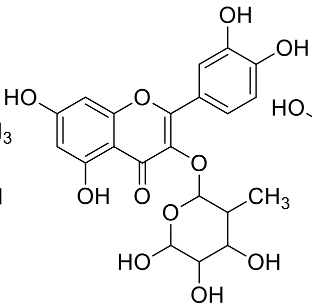
153

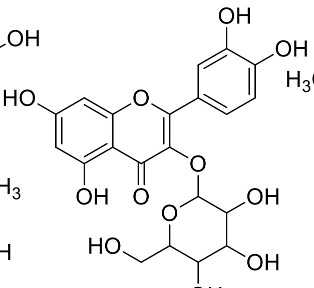
154

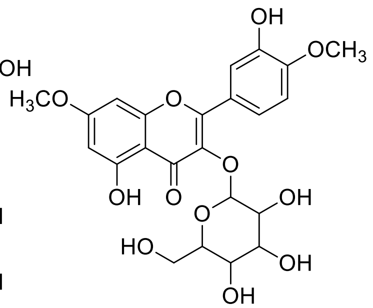
155

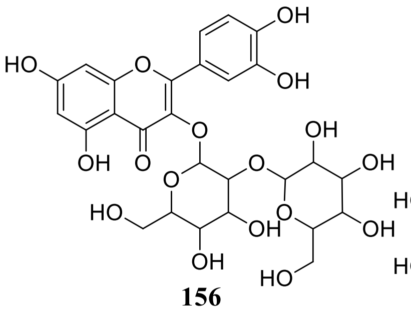
156

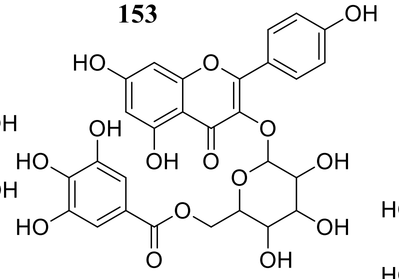
157

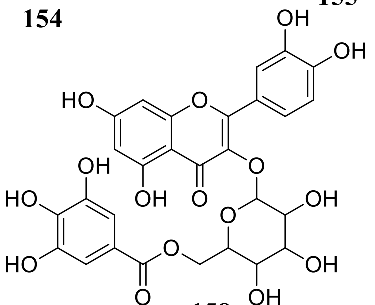
158

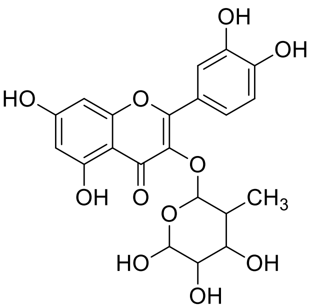
159

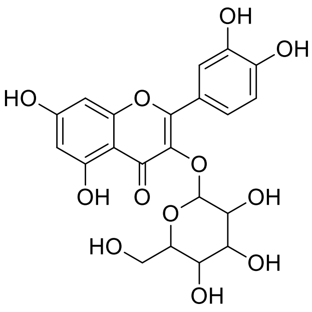
160

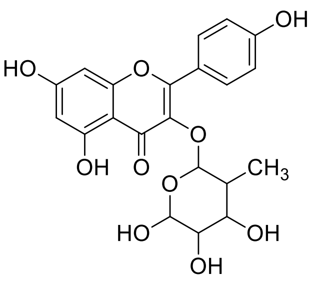
161

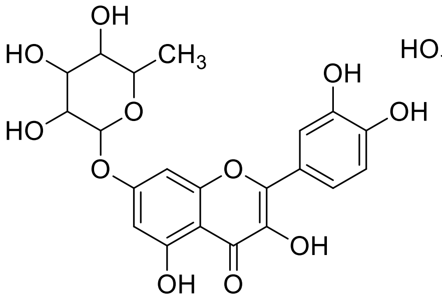

162

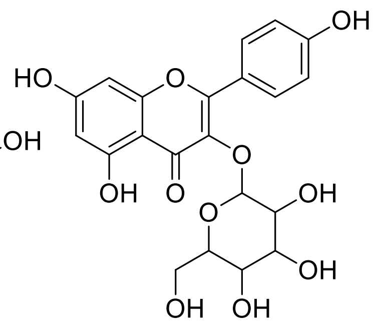

163

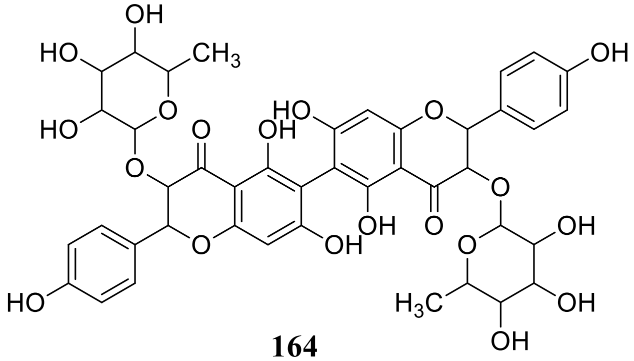

164

Figure S1. Chemical structures of bioflavonoids from genus *Bauhinia.*
